# Foraging strategy as a route for sexual size dimorphism evolution

**DOI:** 10.1002/ece3.70100

**Published:** 2024-11-07

**Authors:** Pedro N. Rocha, Felipe M. Gawryszewski

**Affiliations:** ^1^ Departamento de Zoologia, Evolutionary Ecology Laboratory Universidade de Brasília Brasília Brazil; ^2^ Programa de Pós‐Graduação em Ecologia Universidade de Brasília Brasília Brazil

**Keywords:** body size, crab spider, fecundity selection, luring, sexual selection, sexual size dimorphism, SSD, Thomisidae

## Abstract

Female‐biased sexual size dimorphism stands as a widespread evolutionary pattern. Fecundity selection, favouring larger females with greater reproductive output, is a leading explanation. However, larger body sizes demand greater energy intake, potentially hindering the evolution of extreme female sizes. Thus, the evolution of more lucrative foraging tactics may allow for an increase in size. Hence, coupled with selection against larger males, fecundity selection should result in larger SSD in species with more lucrative foraging strategies. Crab spiders are sit‐and‐wait predators that hunt in several plant substrata. Species that forage on flowers or employ prey‐luring strategies likely have access to higher food intake than other species. We extracted body size measurements of 614 crab spider species from 43 genera and classified them based on their foraging strategy. Our findings show that foraging strategies that provide higher energy input (EFS) result in larger SSD. Statistical estimates indicate that females have a cephalothorax width 91% larger than males in EFS species, compared to 26% larger females than males in non‐EFS species. These differences possibly arise due to larger females and smaller males. The effects on male size reduction might result from scramble competition, whereas the increase in female size is likely due to fecundity selection. These results suggest that the shift towards more lucrative foraging strategies may have been a key event in body size and SSD evolution in crab spiders.

## Graphical abstract

The study reveals a link between hunting strategy and sexual size dimorphism in crab spiders. Female crab spiders that actively hunt prey (floral and lure) are significantly larger than males. This is attributed to the increased energy intake these strategies provide, allowing females to allocate more resources towards reproduction and smaller males, possibly due to stronger scramble competition.

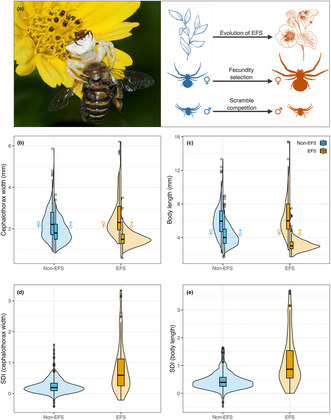

## 1 INTRODUCTION

Sexual size dimorphism (SSD) is the difference in size between the sexes of a given species. The differential equilibrium model suggests that contrasting evolutionary pressures are operating on male and female sizes (Andersson, 1994; Blanckenhorn, 2005; Kuntner & Coddington, 2020). In general, a genetic correlation between females and males is expected. Therefore, selection favouring a larger body size in one sex would also lead to the evolution of a larger body size in the other sex. However, when antagonistic selection pressures occur, the correlation between the male and female size may be lessened, resulting in SSD.

It is commonly accepted that the evolution of male‐biased SSD (larger males than females) is a product of sexual selection through male‐male competition or female choice, as larger males are better competitors for female access or other resources (Andersson, 1994). This pattern is readily observed in endothermic animals such as mammals and birds (Andersson, 1994; Fairbairn et al., 2007). In ectothermic animals, however, female‐biased SSD (larger females than males) is more prevalent.

In *The Descent of Man and Selection in Relation to Sex*, Darwin proposed fecundity selection as the reason why female‐biased SSD evolves in so many animal groups (Darwin, 1871). Fecundity selection assumes that the larger the female, the larger their clutch. This phenomenon has been observed in several animal taxa, such as insects (Honěk, 1993), reptiles (Cox et al., 2003; Shine, 1994), mammals (Fokidis et al., 2007) and spiders (Prenter et al., 1999), and is still considered the primary explanation for female‐biased SSD evolution (but see Pincheira‐Donoso & Hunt, 2017). On insects, for instance, a 1% increase in dry body weight is estimated to result in a 0.95% increase in fecundity (Honěk, 1993). However, there is no clear answer to why female‐biased SSD has evolved in some species rather than others.

Spiders have been a long‐studied group for the evolution of female‐biased SSD. Most spider species are sexually dimorphic, and extreme cases of SDD have evolved; some species with a mean female weight 40 times heavier than the male (Elgar & Fahey, 1996). Overall, the evolution of SSD in spiders appears to have occurred through increases in female size rather than male size reduction (Coddington et al., 1997; Head, 1995; Hormiga et al., 2000; Prenter et al., 1999). Moreover, female‐biased SSD has evolved multiple times within the group (Hormiga et al., 2000). The reasons why are not yet clear, but fecundity selection is the prime candidate (Foellmer & Moya‐Larano, 2007).

A higher dietary intake is needed to sustain larger body sizes. In the orb‐web spider *Trichonephila clavipes*, for instance, females cannot reach sexual maturity with the same diet as the smaller males (Higgins & Goodnight, 2010). On Nephilinae spiders, extreme SSD is correlated with larger aerial webs (Kuntner et al., 2019). On praying mantises, SSD is correlated with flower mimicry, which may increase prey consumption and allow female growth (Svenson et al., 2016). Therefore, a more lucrative foraging strategy may lead to the evolution of larger female body sizes due to fecundity selection.

However, SSD would not evolve if the fecundity selection on females led to larger males due to genetic correlations. In the collared flycatcher (*Ficedula albicollis*), for instance, the absence of SSD is attributed to the high genetic correlation between the sexes, even though the predicted selection would result in females four times larger and males half the size observed (Merila et al., 1998). In spiders, the differential equilibrium model (reviewed in Kuntner & Coddington, 2020) proposes that several evolutionary pressures occur antagonistically on the sexes. In this model, the SSD evolution is the product of opposing selection pressures, which result in a positive net sum for females and a negative sum for males. There are several hypotheses to explain the advantages of smaller spider males. The most prominent are scrambled competition (Moya‐Laraño et al., 2002, 2009), the differential mortality model (Vollrath & Parker, 1992) and sexual cannibalism (Wilder & Rypstra, 2008).

In the scramble competition scenario, there is a fitness advantage for males arriving earlier on non‐choosy females (Foellmer & Moya‐Larano, 2007; Huber, 2005). Therefore, mating occurs in a scrambled competition context, which leads to protandry and, consequently, smaller males (Maklakov et al., 2004; Morbey & Ydenberg, 2001). Further, in spiders, smaller males may move faster and, therefore, would be favoured in this context (‘gravity hypothesis’; Moya‐Laraño et al., 2002). The differential mortality model proposes that males from active hunter spider species would have higher mortality rates when compared to sit‐and‐wait species (Vollrath & Parker, 1992). This would result in lower male density and lessen male‐male competition, consequently removing the potential advantages of larger and costly males. Finally, the sexual cannibalism hypothesis proposes that smaller males are at a lower risk of being cannibalised by females during sexual encounters, which would lead to selection favouring size reduction in males (Wilder & Rypstra, 2008).

**FIGURE 1:**
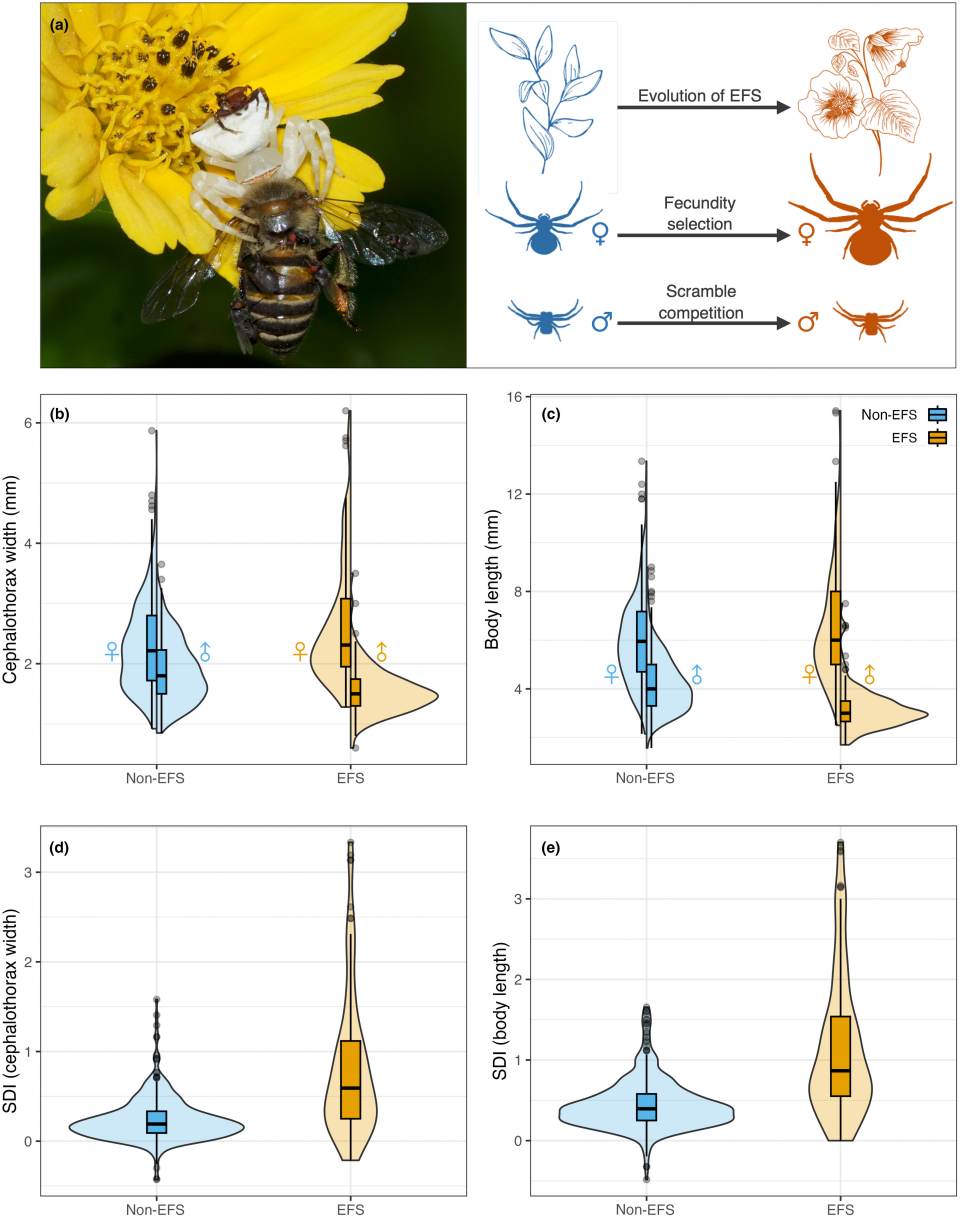
(a) A female of *Thomisus* sp. preying on a bee and the male crab spider on top of her. The scheme shows an evolutionary shift to EFS, leading to an increase in prey consumption and allowing female growth through fecundity selection over evolutionary time. Scramble competition may explain no change or a decrease in male size. These processes should lead to larger sexual size dimorphism in EFS species. Raw data distribution of (b) cephalothorax width (*N* = 496 species) and (c) body length (*N* = 561 species) of females and males of crab spider species based on the occurrence of exploitative foraging strategies. Sexual Size Dimorphism Index (SDI) for (d) cephalothorax width and body length (e) by the occurrence of exploitative foraging strategies (EFS). Photo by Vineeth Vengolis used under CC BY‐SA 4.0.

Crab spiders (Thomisidae) are sit‐and‐wait predators that wait for prey on different plant structures and leaf litter. The most commonly known crab spiders hunt on flowers. Compared to other substrata, flower crab spiders likely have access to a higher and/or more regular prey intake due to the attractiveness of flowers (Figure 1a). Females of *Misumena vatia*, a flower‐foraging crab spider, regularly prey on *Apis mellifera* bees, an abundant and large prey item. Further, females of *Misumena* occasionally capture the even larger *Bombus* bees, which provide higher energy consumption, causing increased growth rate, weight and number of eggs (Fritz & Morse, 1985). Moreover, some crab spider genera employ prey‐luring strategies. UV‐reflective female flower crab spiders are known to attract bees (Cheng et al., 2006; Heiling et al., 2003; Heiling, Cheng, et al., 2005a; Heiling, Chittka, et al., 2005b; Herberstein et al., 2009; Welti et al., 2016). The contrast between a UV‐reflecting *Thomisus spectabilis* female and a non‐UV‐reflective flower increases the probability of a bee visiting a flower that has a spider present (Heiling et al., 2003; Heiling, Cheng, et al., 2005a) and is associated with a better female body condition (Gawryszewski et al., 2012).

Other female crab spiders mimic a foraging resource their prey is interested in (aggressive mimicry). Female spiders of the *Epicadus* genus have a modified abdomen that resembles flowers. Bees are commonly attracted to these spiders even when the spider is not sitting on a flower (Vieira et al., 2017). Similarly, species in the genus *Phrynarachne* resemble bird excrement, which was formerly perceived solely as a strategy to avoid predation. However, *Phrynarachne*'s masquerade has been shown to attract dipterans and hymenopterans at a higher rate than a plain leaf; moreover, visually manipulated spiders attracted significantly fewer insects than unmanipulated spiders (Yu et al., 2022).

**FIGURE 2:**
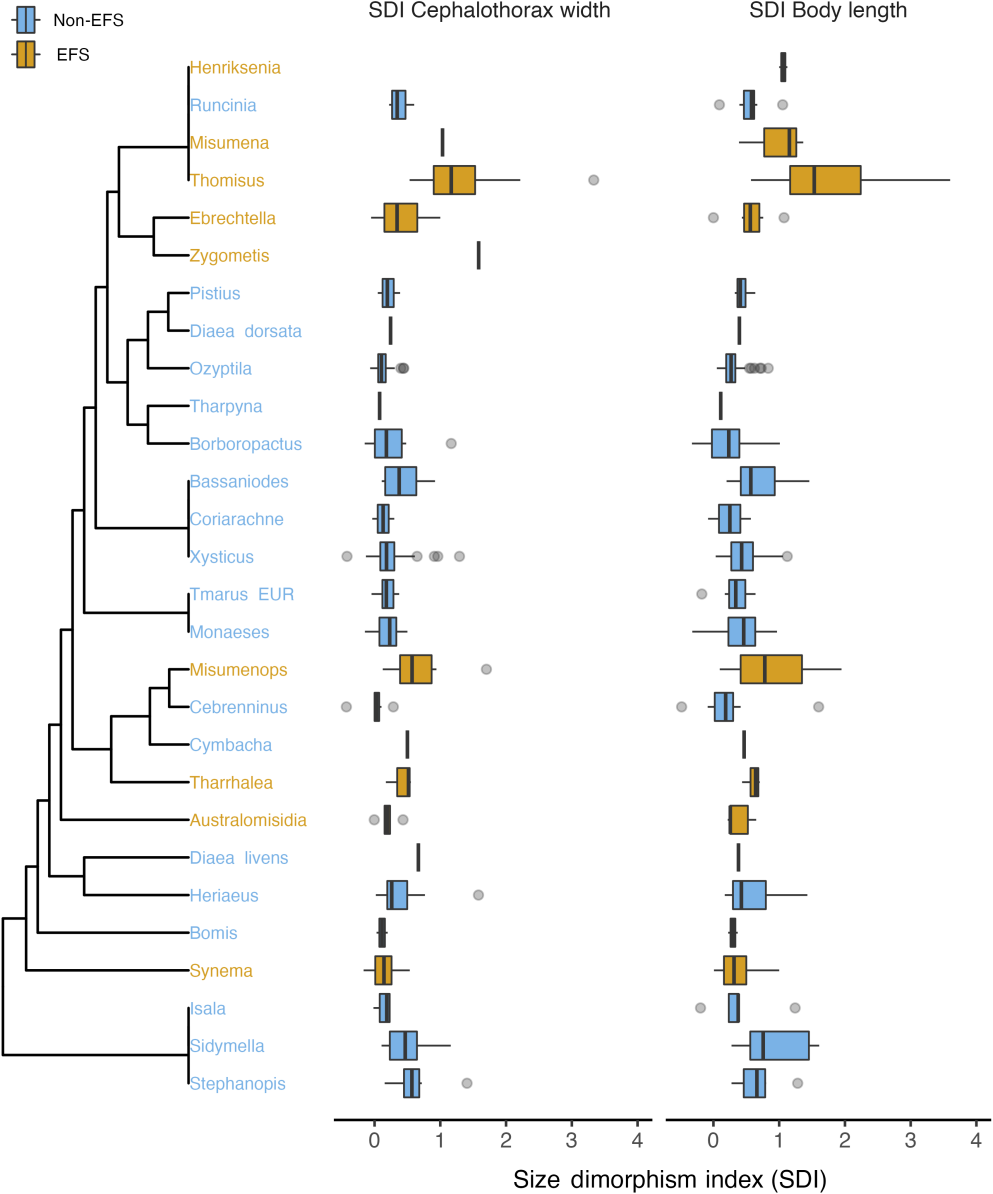
Backbone phylogeny of crab spiders and the distribution of Sexual Size Dimorphism of values of species within each clade for groups that employ Exploitative Foraging Strategies (EFS) and do not (non‐EFS).

Phylogenetic analyses place crab spider genera that employ aggressive mimicry (*Epicadus* and *Phrynarachne*) apart from each other and flower‐dwelling species (Benjamin, 2011; Machado & Teixeira, 2021). Further, flower dwelling has evolved independently at least five times within crab spiders (Gawryszewski et al., 2017; Figure 2). The independent evolution of these exploitative foraging strategies (EFS; hunting on flowers and prey luring) might provide a higher prey capture by exploiting prey foraging behaviour and, consequently, influence the evolution of body sizes and SSD. The evolution of EFS might provide a pathway to surpass the energetic limitations of larger sizes and, in turn, allow for fecundity selection to increase female size in crab spiders. Therefore, we hypothesise that EFS species will have larger females than non‐EFS species. Furthermore, selection against larger males should result in no change between EFS and non‐EFS species. This would lead to a reduction in the genetic correlation of male and female sizes and, consequently, the evolution of increased SSD in EFS species (Figure 1b). We tested these ideas in a comparative framework after gathering data from 614 species within 43 genera worldwide distributed.

## 2 METHODS

### 2.1 Data collection

To evaluate the size evolution of each sex, we extracted the cephalothorax width and body length from taxonomical papers listed in the World Spider Catalog (2021). In total, we collected size data from 614 species within 47 genera. We only included species that had size measurements for both sexes and, when possible, always chose the most recent article that contained measurements for both sexes. We used the measurements of the type specimens if provided, or the mean if the article provided a size range. Carapace width (CW) is considered a better measurement of size than body length (BL) because the latter might be confounded with body condition (Foellmer & Fairbairn, 2005; Foellmer & Moya‐Larano, 2007). However, body length is more readily available in taxonomical works; therefore, we extracted both.

We extracted data from genera present in five different crab spider phylogenies (Benjamin et al., 2008; Benjamin & Clayton, 2016; Gawryszewski et al., 2017; Machado & Teixeira, 2021; Vieira, 2015). We chose to select genera from phylogenies to reduce taxonomical inconsistencies. This phylogenetic diversity resulted in worldwide distributed species encompassing all major biogeographical areas. According to the scientific literature and other sources, we classified them as Exploitative Foraging Strategy (EFS) spiders that forage on flowers or employ aggressive mimicry (Table S1). We did not classify genera that mimic ants (e.g. *Aphantochilus*) as EFS because those genera likely have their size constrained by the size of the model species. Therefore, females cannot outgrow the ants it mimics, as this might result in a loss of the mimicry itself. We performed a genus‐level classification, as detailed species‐level natural history data are scarce and spiders have little intra‐generic variation in foraging strategies. We excluded from the analyses genera without foraging behaviour data.

### 2.2 Statistical analysis

We analysed the data through multivariate Bayesian linear mixed models using either the genera or phylogenies to account for the effect of phylogenetic history on statistical estimates. In the genus analysis, the genera entered into the group‐level (random) structure (a taxonomic mixed model). For the phylogenetic analysis, we used the majority‐rule consensus tree recovered from the phylogenetic analyses, based on three genetic markers, of Gawryszewski et al. (2017). The other phylogenetic trees were not suited to our analysis due to a small number of EFS species (Benjamin et al., 2008; Benjamin & Clayton, 2016; Machado & Teixeira, 2021; Vieira, 2015). We ran both the taxonomic and the phylogenetic models because not all genera measured were present in the phylogenetic tree. We updated taxonomic names based on the World Spider Catalogue (2021) and transformed the tree into a backbone genus‐level phylogeny. For the analysis, we included both the genus‐level phylogeny and the genera as group‐level effects (a phylogenetic and taxonomic mixed model *sensu* Hadfield & Nakagawa, 2010). We pruned genera for which we did not have foraging mode data and converted clades with paraphyletic genera to zero‐length branches. The tree also indicated two polyphyletic genera, *Diaea* and *Tmarus* (Gawryszewski et al., 2017). Both *Diaea* species in the phylogeny have a Paleartic distribution (*D. livens* and *D. dorsata*). For these, we only included data for the species present in the phylogeny and treated them as separated genera. *Tmarus* species were split into two clades composed of Australian and Paleartic species. We treated each of these clades as separate genera and only included data from species occurring in these regions.

To evaluate the evolution of male and female body sizes, we used male and female cephalothorax width (CW) or male and female body length (BL) as response variables and foraging strategy (EFS or non‐EFS) as the explanatory variable. In all models, cephalothorax width was log‐transformed and body length was square root transformed. For the ease of comparison with the Sexual Size Dimorphism Index (below), we used the posterior draws from the cephalothorax width and body length statistical models to calculate a proportional index of difference (DI) between EFS and non‐EFS species so that a value of zero indicates no difference in size, positive values indicate that EFS spiders are larger than non‐EFS spiders, and negative values indicate non‐EFS spiders are larger than EFS spiders (e.g. a value of +0.5 for female cephalothorax width corresponds to 50% larger EFS females than non‐EFS females). The calculation of the index does not alter the statistical estimates; it simply converts an estimated difference in millimetres to a proportion.

For the genus analysis, we had a sample size of 479 species and 37 genera (12 EFS and 25 non‐EFS) for the cephalothorax width and 554 species and 42 genera (14 EFS and 28 non‐EFS) for body length. For the model incorporating the phylogeny, we had 361 species and 26 genera (8 EFS and 18 non‐EFS) for cephalothorax width and 391 species and 26 genera (8 EFS and 18 non‐EFS) for body length.

To evaluate the evolution of sexual size dimorphism, we used female and male sizes to calculate the Sexual Size Dimorphism Index (SDI) of cephalothorax width and body length, as follows (Lovich & Gibbons, 1992):

If the female is the larger sex:

(1) $$\mathrm{SDI}=\frac{{\text{Size}}_{\text{female}}}{{\text{Size}}_{\text{male}}}-1$$

If the male is the larger sex:

$$\mathrm{SDI}=\left(\frac{{\text{Size}}_{\text{male}}}{{\text{Size}}_{\text{female}}}-1\right)\times \left(-1\right)$$

Therefore, a value of zero indicates no difference in the size of females and males; positive values indicate that females are larger than males; and negative values indicate that males are larger than females (e.g. a value of +0.5 indicates that females are 50% larger than males). Sample sizes are the same as in the CW and BL models described above. Model checks indicated different variances between EFS and non‐EFS groups. Therefore, we included a model parameter to estimate variances (sigma) for each group separately.

We ran Bayesian models in R (v.4.3.1; R Core Team, 2023) using the package brms (v2.20.1; Bürkner, 2018), which implements Bayesian models in Stan (Carpenter et al., 2017). We used weakly informative priors, akin to Goodrich et al. (2023; see Table S2 for priors). For each model, we ran four independent chains for 6000 iterations, 3000 as a warm‐up (burn‐in), in a total of 12,000 sampled iterations. We evaluated model fits by checking chain convergence, the presence of transitions with diverging errors, visual posterior predictive checks and leave‐one‐out cross‐validation.

## 3 RESULTS

**FIGURE 3:**
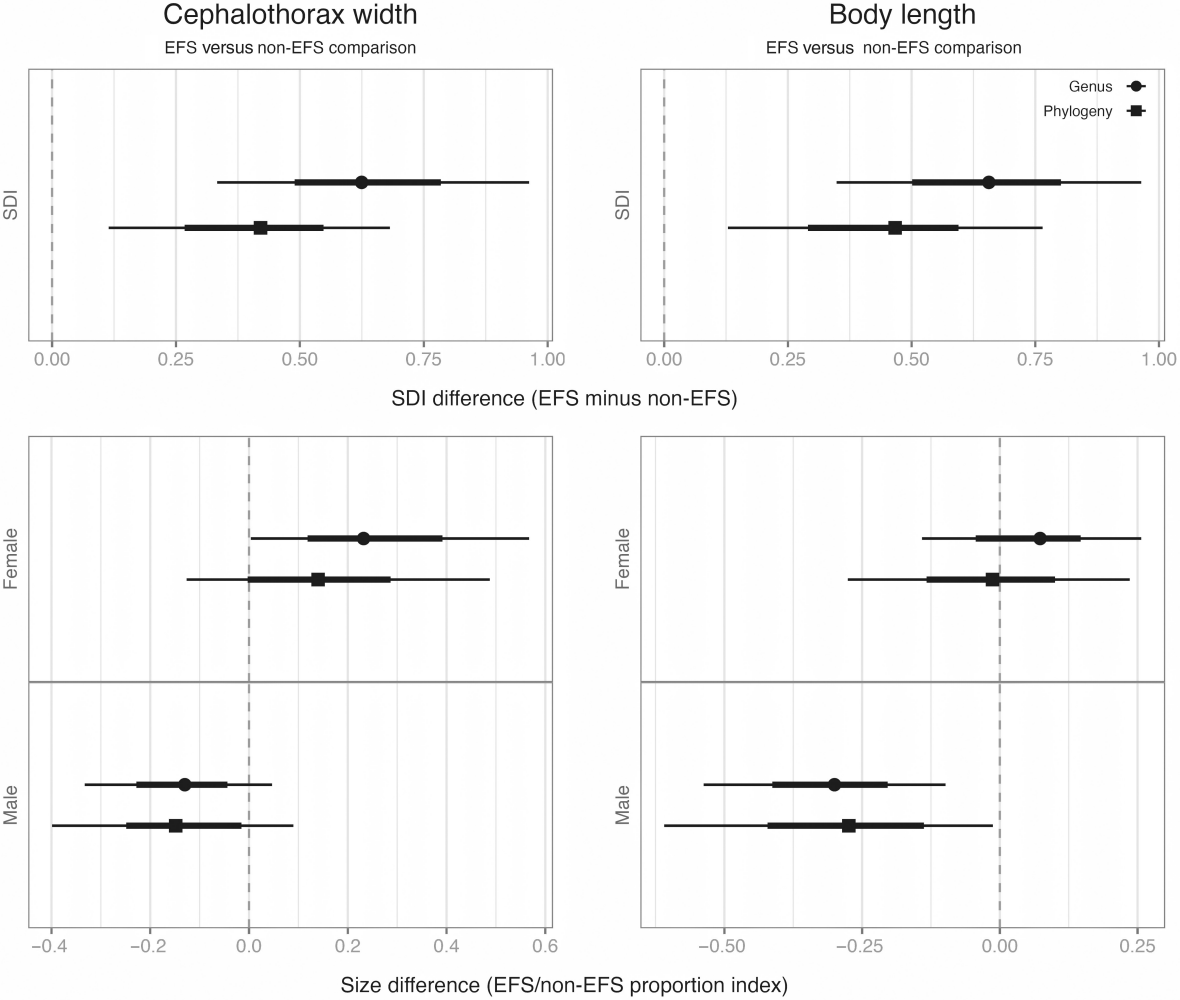
Bayesian statistical comparison between EFS and non‐EFS crab spiders for the Sexual Size Dimorphism Index (SDI), as well as female and male cephalothorax width and body length. The cephalothorax and body length comparison are a proportion index so that, similar to SDI, positive values indicate larger EFS females or males than non‐EFS females or males. Statistical models using either the genera or the phylogeny as group‐level factors. Points denote the mode; thick and thin lines denote 67% and 95% highest density credible intervals.

The employment of EFS is associated with larger body sizes in females and smaller sizes in males, which resulted in a larger sexual size dimorphism in EFS spiders for both cephalothorax width and body length (Figures 1 and 2). Both the genus and phylogenetic statistical models estimate that EFS spiders have ca. 0.5 points of Sexual Size Dimorphism Index (SDI) more than non‐EFS spiders (Figure 3, top row). The statistical estimates for SDI indicate that in EFS species, females have a cephalothorax width ca. 60%–90% larger than males, whereas in a non‐EFS species, females are expected to have a cephalothorax width ca. 25% larger than males (genus model: EFS = 0.91 [0.64, 1.17], non‐EFS = 0.26 [0.10, 0.43]; phylogeny model: EFS = 0.65 [0.34, 0.97], non‐EFS = 0.27 [−0.02, 0.49]; mode and 95% highest density credible interval). For body length, the statistical estimates are 85%–110% larger EFS females than males and ca. 45% larger non‐EFS females than males (genus model: EFS = 1.09 [0.84, 1.36], non‐EFS = 0.46 [0.29, 0.62]; phylogeny model: EFS = 0.86 [0.52, 1.20], non‐EFS = 0.44 [0.11, 0.66]; mode and 95% highest density credible interval). Tables with model coefficients are provided in the Supporting Information.

The SDI of cephalothorax width and body length are similar. However, they may arise for different reasons. For cephalothorax width, the statistical estimates from both models suggest that EFS species have larger females and smaller males than non‐EFS species (Figure 3, bottom row). On the other hand, for body length, the point estimates suggest EFS and non‐EFS females of similar size but smaller EFS males than non‐EFS (Figure 3, bottom row). However, uncertainty around these estimates only allows for a clear difference between the cephalothorax width of EFS and non‐EFS females and the body length of EFS and non‐EFS males (Figure 3, bottom row; tables with model coefficients are provided in the Supporting Information).

## 4 DISCUSSION

Our results clearly indicate an association between Exploitative Foraging Strategies (EFS) and body size evolution. Sexual size dimorphism is significantly more prominent in EFS than in non‐EFS crab spiders. For the evolution of SSD to occur, it is necessary that the males do not increase at the same rate as females (Andersson, 1994; Blanckenhorn, 2005). In crab spiders, our results suggest that the SSD may arise from both larger females and smaller males in EFS species.

Fecundity selection is evoked as the primary selective pressure for increased female size in most animal groups with female‐biased SSD (Fairbairn et al., 2007). Several studies have provided strong evidence for the reproductive advantages of larger female sizes (Honěk, 1993; Prenter et al., 1999). Our work provides further support in favour of fecundity selection. It suggests a route for the increase in female body size, as the exploitative foraging strategies are a way to surpass the energetic limitations of larger sizes. Therefore, in crab spiders, the evolutionary increase in female size could be restrained by dietary reasons.

Although we did not predict the size reduction in ESF males, our models have shown that males of EFS species have significantly smaller body lengths than those who employ other foraging strategies. Foraging strategies have already been suggested as the cause of SSD in spiders due to male dwarfism. Active hunters would have higher mortality rates during mate search, resulting in lessened male‐male competition and smaller males (Vollrath & Parker, 1992). However, phylogenetic comparisons and experimental data have shown no association between active and sit‐and‐wait foraging strategies and SSD occurrence or female body size (Prenter et al., 1997, 1998; Walker & Rypstra, 2003; Wolff et al., 2022).

In crab spiders, increased selective advantages of protandry might play a role in the evolution of smaller EFS males. If female EFS spiders depend on flowers for foraging and survival, those species likely have an aggregated distribution due to the aggregated nature of flowers. Therefore, males with shorter development and consequently smaller size may reach sites with rich female density with little to no male competition (i. e. mate opportunity hypothesis; Morbey & Ydenberg, 2001). Moreover, smaller males may have an advantage due to higher mobility (gravity hypothesis; Moya‐Laraño et al., 2002). However, the gravity hypothesis has failed to hold itself true in experimental trials with no effect of size on climbing speed (Brandt & Andrade, 2007; Prenter et al., 2010; Quiñones‐Lebrón et al., 2019) or males having an optimum body size for climbing (Foellmer et al., 2011; Moya‐Laraño et al., 2009). Furthermore, sexual cannibalism might play a role in the SSD evolution of Thomisids. *Misumena vatia*, an EFS species, is known to prey on males (Morse, 2004; Morse & Hu, 2004). However, to our knowledge, there is little data on non‐EFS or other EFS species mating behaviour to propose an evolutionary pattern of male size constraints due to sexual cannibalism on Thomisidae spiders.

In addition to an increase in size, the evolution of EFS may also be linked to alterations in body shape. EFS males tend to exhibit a smaller cephalothorax width and body length compared to non‐EFS males. Conversely, among females, the discrepancy in cephalothorax width between EFS and non‐EFS spiders appears more pronounced than the difference in body length. This suggests a potential correlation between EFS evolution and alterations in female body shape. In orb‐web spiders, differences in female body shape across species have been associated with their likelihood of sun exposure (Ferreira‐Sousa et al., 2021). In crab spiders, distinct body shapes might result from selection pressures related to mobility or camouflage, differing based on the substrates inhabited by EFS and non‐EFS species (e.g. flowers vs. leaf litter). In Araneae, an overall change in cephalothorax shape is not correlated with foraging strategy (Wolff et al., 2022). Nonetheless, a localised analysis of thomisids may reveal a pattern between EFS and non‐EFS species.

Furthermore, the association between exploitative foraging strategies and SSD might result from other causes. Our hypothesis suggests that the evolution of alternative foraging strategies enables organisms to access increased prey consumption and achieve larger sizes, thus causing SSD. On the other hand, larger Sexual Size Dimorphism evolution on EFS species might also be a product of other behavioural changes associated with EFS, such as lower mobility in females and the aforementioned protandry in males.

In other groups, the evolution of EFS may also lead to larger SSD. For instance, the spider genera *Mastophora* have some of the most striking cases of female‐biased SSD. *Mastophora* has a unique foraging strategy in which they mimic virgin female moths' pheromones, which in turn attract male moths (Eberhard, 1977). This specialised foraging strategy might increase prey consumption, allowing for female size increase through fecundity selection. Nonetheless, SSD in *Mastophora* might be the result of protandry, as males go through only one to two instars before sexual maturity (Eberhard, 1980) or even emerge as adults (Gertsch, 1955). Similarly, the light‐emitting modified dorsal fin of anglerfishes might increase the female probability of catching prey (Pietsch, 2009), contributing to the evolution of extreme SSD in this group.

In conclusion, body size and SSD evolution are associated with foraging strategies in crab spiders. Foraging strategies that supply higher energy intake may allow for increased female growth through fecundity selection, while other less profitable foraging strategies may limit female body size. Also, foraging strategies seem to affect male sizes, as males in EFS species were smaller than in species that do not employ EFS. The increased female size and reduced male size result in larger SSD on EFS crab spiders. Therefore, the evolution of more lucrative foraging strategies appears to have been a key event for the increase in sexual size dimorphism in crab spiders.

## AUTHOR CONTRIBUTIONS

**Pedro N. Rocha:** Conceptualization (equal); data curation (lead); formal analysis (supporting); investigation (lead); methodology (equal); writing – original draft (lead); writing – review and editing (equal). **Felipe M. Gawryszewski:** Conceptualization (equal); formal analysis (lead); methodology (equal); project administration (lead); resources (lead); supervision (lead); validation (lead); writing – original draft (supporting); writing – review and editing (equal).

## FUNDING INFORMATION

P.N.R. received a scholarship from Coordenação de Aperfeiçoamento de Pessoal de Nível Superior (CAPES).

## CONFLICT OF INTEREST STATEMENT

We declare no competing interests.

## Supporting information


Table S1

Table S2

## Data Availability

Data sources are provided in Table S1. Raw data and code are available at: https://figshare.com/s/f848b6423167ea540376.
