# Supplementary material for: Foraging strategy as a route for sexual size dimorphism evolution
Source: Ecol Evol. 2024 Nov 7;14(11):e70100. doi: 10.1002/ece3.70100 (PMC11542996; doi:10.1002/ece3.70100)
Supplement: Supplementary file 1 — Table S1 [file ECE3-14-e70100-s002.docx]

**Supplementary Table 1.** All genera measured during the data collection with Exploitative Foraging Strategy classification, the source of the measurements for male and female sizes and the source of the foraging strategy classification.

| **Genus** | **EFS** | **EFS Source** | **Species** | **Source size female** | **Source size male** |
| --- | --- | --- | --- | --- | --- |
| Acentroscelus | non-EFS | (Vieira, 2015) | A. albipes | (Rinaldi, 1983) | (Rinaldi, 1983) |
|  |  |  | A. guianensis | (Keyserling, 1880) | (Keyserling, 1880) |
|  |  |  | A. peruvianus | (Keyserling, 1880) | (Keyserling, 1880) |
| Aphantochilus | non-EFS | (Cushing, 2012) | A. cambridgei | (Canals, 1933) | (Canals, 1933) |
| Australomisidia | EFS | (Gawryszewski et al., 2017) | A. cruentata | (Szymkowiak, 2014) | (Szymkowiak, 2014) |
|  |  |  | A. ergandros | (Szymkowiak, 2014) | (Szymkowiak, 2014) |
|  |  |  | A. inornata | (Szymkowiak & Dymek, 2012) | (Szymkowiak & Dymek, 2012) |
|  |  |  | A. kangarooblaszaki | (Szymkowiak, 2008) | (Szymkowiak, 2008) |
|  |  |  | A. socialis | (Szymkowiak, 2014) | (Szymkowiak, 2014) |
| Bassaniodes | non-EFS | (Jocqué, 1993) | B. bliteus | (Levy, 1976) | (Levy, 1976) |
|  |  |  | B. bufo | (Simon, 1875) | (Levy, 1976) |
|  |  |  | B. canariensi | (Wunderlich, 1987) | (Wunderlich, 1987) |
|  |  |  | B. caperatus | (Levy, 1976) | (Levy, 1976) |
|  |  |  | B. clavulus | (Wunderlich, 1987) | (Wunderlich, 1987) |
|  |  |  | B. cribratus | (Kritscher, 1962) | (Kritscher, 1962) |
|  |  |  | B. egenus | (Simon, 1886b) | (Simon, 1886b) |
|  |  |  | B. fienae | (Jocqué, 1993) | (Jocqué, 1993) |
|  |  |  | B. lalandei | (Levy, 1976) | (Levy, 1999) |
|  |  |  | B. loeffleri | (Marusik & Logunov, 1990) | (Marusik & Logunov, 1990) |
|  |  |  | B. ovcharenkoi | (Marusik & Logunov, 1990) | (Marusik & Logunov, 1990) |
|  |  |  | B. pseudorectilineus | (Demir et al., 2008) | (Demir et al., 2008) |
|  |  |  | B. rectilineus | (Levy, 1976) | (Levy, 1976) |
|  |  |  | B. robustus | (Almquist, 2006) | (Almquist, 2006) |
|  |  |  | B. tristrami | (Tripathi et al., 2023) | (Tripathi et al., 2023) |
|  |  |  | B. turlan | (Marusik & Logunov, 1995) | (Marusik & Logunov, 1990) |
|  |  |  | B. ulkan | (Esyunin et al., 2007) | (Marusik & Logunov, 1990) |
|  |  |  | B. xizangensis | (L. R. Tang & Song, 1988) | (L. R. Tang & Song, 1988) |
| Bomis | non-EFS | (Gawryszewski et al., 2017) | B. hippoponoi | (Szymkowiak & Królikowska, 2017) | (Szymkowiak & Królikowska, 2017) |
|  |  |  | B. larvata | (Szymkowiak & Królikowska, 2017) | (Szymkowiak & Królikowska, 2017) |
| Borboropactus | non-EFS | (Barrion & Litsinger, 1995; Yin et al., 2012) | B. bituberculatus | (G. Tang & Li, 2010a) | (Song, 1993) |
|  |  |  | B. brevidens | (G. Tang & Li, 2010a) | (G. Tang & Li, 2010a) |
|  |  |  | B. cinerascens | (Barrion & Litsinger, 1995) | (Barrion & Litsinger, 1995) |
|  |  |  | B. edentatus | (G. Tang & Li, 2010a) | (G. Tang & Li, 2010a) |
|  |  |  | B. jiangyong | (Meng et al., 2019) | (Meng et al., 2019) |
|  |  |  | B. nyerere | (Benjamin, 2011) | (Benjamin, 2011) |
| Cebrenninus | non-EFS | (Benjamin, 2016) | C. berau | (Benjamin, 2016) | (Benjamin, 2016) |
|  |  |  | C. kalawitana | (Benjamin, 2016) | (Benjamin, 2016) |
|  |  |  | C. magnus | (G. Tang & Li, 2010) | (G. Tang & Li, 2010) |
|  |  |  | C. phaedrae | (Benjamin, 2016) | (Benjamin, 2016) |
|  |  |  | C. rugosus | (Benjamin, 2016) | (Benjamin, 2016) |
|  |  |  | C. schawalleri | (Benjamin, 2016) | (Benjamin, 2016) |
|  |  |  | C. srivijaya | (Benjamin, 2011) | (Benjamin, 2011) |
|  |  |  | C. striatipes | (Benjamin, 2016) | (Benjamin, 2016) |
| Coenypha | non-EFS | (Machado & Teixeira, 2021) | C. ditissima | (Simon, 1887) | (de Mello-Leitão, 1951) |
| Coriarachne | non-EFS | (Almquist, 2006; Dondale & Redner, 1978; Gawryszewski et al., 2017) | C. brunneipes | (Dondale & Redner, 1978) | (Dondale & Redner, 1978) |
|  |  |  | C. depressa | (Almquist, 2006) | (Almquist, 2006) |
|  |  |  | C. fulvipes | (Ono, 1988) | (Ono, 1988) |
| Cymbacha | non-EFS | (Gawryszewski et al., 2017) | C. saucia | (Thorell, 1881) | (Thorell, 1881) |
| Diaea | non-EFS | (Gawryszewski et al., 2017) | D. albicincta | (de Lessert, 1919) | (de Lessert, 1919) |
|  |  |  | D. delata | (Karsch, 1880) | (Karsch, 1880) |
|  |  |  | D. dorsata | (Almquist, 2006) | (Almquist, 2006) |
|  |  |  | D. gyoja | (Ono, 1985a) | (Ono, 1985a) |
|  |  |  | D. limbata | (Kulczynski, 1911) | (Kulczynski, 1911) |
|  |  |  | D. livens | (Buchar & Thaler, 1984) | (Buchar & Thaler, 1984) |
|  |  |  | D. mikhailovi | (Guo & Zhang, 2014) | (Guo & Zhang, 2014) |
|  |  |  | D. mutabilis | (Kulczyński, 1901) | (Kulczyński, 1901) |
|  |  |  | D. osmanii | (Zamani & Marusik, 2017) | (Zamani & Marusik, 2017) |
|  |  |  | D. puncta | (Millot, 1942) | (Millot, 1942) |
|  |  |  | D. sphaeroides | (Bryant, 1933) | (Bryant, 1935) |
|  |  |  | D. subdola | (Yin et al., 2012) | (Yin et al., 2012) |
|  |  |  | D. suspiciosa | (Song & Hu, 1986) | (Song & Hu, 1986) |
| Ebrechtella | EFS | (S. T. Kim & Lee, 2012) | E. juwangensis | (Seo, 2015) | (Seo, 2015) |
|  |  |  | E. margaritacea | (Simon, 1909) | (Simon, 1909) |
|  |  |  | E. pseudovatia | (Song & Zhu, 1997) | (Song & Zhu, 1997) |
|  |  |  | E. sufflava | (Marusik, 1993) | (Marusik, 1993) |
|  |  |  | E. timida | (Thorell, 1887) | (Thorell, 1895) |
|  |  |  | E. tricuspidatus | (Yin et al., 2012) | (Yin et al., 2012) |
|  |  |  | E. xinjiangensis | (Hu & Wu, 1989) | (Hu & Wu, 1989) |
| Epicadinus | non-EFS | (Prado et al., 2018) | E. biocellatus | (Prado et al., 2018) | (Prado et al., 2018) |
|  |  |  | E. spinipes | (Prado et al., 2018) | (Prado et al., 2018) |
|  |  |  | E. trispinosus | (Prado et al., 2018) | (Prado et al., 2018) |
|  |  |  | E. villosus | (Prado et al., 2018) | (Prado et al., 2018) |
| Epicadus | EFS | (Vieira et al., 2017) | E. caudatus | (Machado et al., 2015) | (Machado et al., 2015) |
|  |  |  | E. dimidiaster | (Machado et al., 2018) | (Machado et al., 2018) |
|  |  |  | E. granulatus | (Machado et al., 2018) | (Machado et al., 2018) |
|  |  |  | E. heterogaster | (Silva-Moreira & Machado, 2016) | (Silva-Moreira & Machado, 2016) |
|  |  |  | E. rubripes | (Silva-Moreira & Machado, 2016) | (Silva-Moreira & Machado, 2016) |
|  |  |  | E. taczanowskii | (Machado et al., 2018) | (Machado et al., 2018) |
|  |  |  | E. tigrinus | (Machado et al., 2018) | (Machado et al., 2018) |
|  |  |  | E. trituberculatus | (Silva-Moreira & Machado, 2016) | (Silva-Moreira & Machado, 2016) |
|  |  |  | E. tuberculatus | (Machado et al., 2015) | (Machado et al., 2015) |
| Epidius | non-EFS | (Joseph & Ambalaparambil, 2017) | E. armatus | (G. Tang et al., 2009) | (G. Tang & Li, 2010b) |
|  |  |  | E. binotatus | (Simon, 1897) | (de Lessert, 1930) |
|  |  |  | E. floreni | (Benjamin, 2017) | (Benjamin, 2017) |
|  |  |  | E. gongi | (Song & Kim, 1992) | (Song & Kim, 1992) |
|  |  |  | E. longimanus | (Benjamin, 2017) | (Benjamin, 2017) |
|  |  |  | E. mahavira | (Benjamin, 2017) | (Benjamin, 2017) |
|  |  |  | E. parvati | (Benjamin, 2000) | (Benjamin, 2000) |
|  |  |  | E. rubropictus | (Yin et al., 1999) | (Yin et al., 1999) |
| Geraesta | Unknown | - | G. congoensis | (Benjamin, 2015) | (Benjamin, 2015) |
|  |  |  | G. mkwawa | (Benjamin, 2011) | (Benjamin, 2011) |
|  |  |  | G. octolobata | (Simon, 1886a) | (Benjamin, 2015) |
| Henriksenia | EFS | (Chua & Lim, 2012) | H. hilaris | (Barrion & Litsinger, 1995) | (Barrion & Litsinger, 1995) |
|  |  |  | H. nepenthicola | (Fage, 1928) | (Fage, 1928) |
| Heriaeus | non-EFS | (Gawryszewski et al., 2017; van Niekerk & Dippenaar-Schoeman, 2013) | H. algericus | (Loerbroks, 1983) | (Loerbroks, 1983) |
|  |  |  | H. allenjonesi | (van Niekerk & Dippenaar-Schoeman, 2013) | (van Niekerk & Dippenaar-Schoeman, 2013) |
|  |  |  | H. antoni | (van Niekerk & Dippenaar-Schoeman, 2013) | (van Niekerk & Dippenaar-Schoeman, 2013) |
|  |  |  | H. buffoni | (Loerbroks, 1983) | (Loerbroks, 1983) |
|  |  |  | H. buffonopsis | (Loerbroks, 1983) | (Loerbroks, 1983) |
|  |  |  | H. concavus | (G. Tang & Li, 2010b) | (G. Tang & Li, 2010b) |
|  |  |  | H. convexus | (G. Tang & Li, 2010b) | (G. Tang & Li, 2010b) |
|  |  |  | H. copricola | (van Niekerk & Dippenaar-Schoeman, 2013) | (van Niekerk & Dippenaar-Schoeman, 2013) |
|  |  |  | H. crassispinus | (van Niekerk & Dippenaar-Schoeman, 2013) | (van Niekerk & Dippenaar-Schoeman, 2013) |
|  |  |  | H. foordi | (van Niekerk & Dippenaar-Schoeman, 2013) | (van Niekerk & Dippenaar-Schoeman, 2013) |
|  |  |  | H. graminicola | (Loerbroks, 1983) | (Loerbroks, 1983) |
|  |  |  | H. hirtus | (Loerbroks, 1983) | (Loerbroks, 1983) |
|  |  |  | H. horridus | (Loerbroks, 1983) | (Loerbroks, 1983) |
|  |  |  | H. maurusius | (Loerbroks, 1983) | (Loerbroks, 1983) |
|  |  |  | H. mellotteei | (Loerbroks, 1983) | (Loerbroks, 1983) |
|  |  |  | H. numidicus | (Loerbroks, 1983) | (Loerbroks, 1983) |
|  |  |  | H. oblongus | (Mcheidze, 2014) | (Mcheidze, 2014) |
|  |  |  | H. orientalis | (Loerbroks, 1983) | (Loerbroks, 1983) |
|  |  |  | H. peterwebbi | (van Niekerk & Dippenaar-Schoeman, 2013) | (van Niekerk & Dippenaar-Schoeman, 2013) |
|  |  |  | H. pilosus | (Loerbroks, 1983) | (Loerbroks, 1983) |
|  |  |  | H. setiger | (Loerbroks, 1983) | (Loerbroks, 1983) |
|  |  |  | H. simoni | (Loerbroks, 1983) | (Loerbroks, 1983) |
|  |  |  | H. spinipalpus | (Loerbroks, 1983) | (Loerbroks, 1983) |
|  |  |  | H. transvaalicus | (van Niekerk & Dippenaar-Schoeman, 2013) | (van Niekerk & Dippenaar-Schoeman, 2013) |
|  |  |  | H. xanderi | (van Niekerk & Dippenaar-Schoeman, 2013) | (van Niekerk & Dippenaar-Schoeman, 2013) |
|  |  |  | H. xinjiangensis | (Liang et al., 1991) | (Liang et al., 1991) |
|  |  |  | H. zanii | (van Niekerk & Dippenaar-Schoeman, 2013) | (van Niekerk & Dippenaar-Schoeman, 2013) |
|  |  |  | H. zhalosni | (Komnenov, 2017) | (Komnenov, 2017) |
| Isala | non-EFS | (Machado, Teixeira, et al., 2019) | I. arenata | (Machado, Teixeira, et al., 2019) | (Machado, Teixeira, et al., 2019) |
|  |  |  | I. cambridgei | (Machado, Teixeira, et al., 2019) | (Machado, Teixeira, et al., 2019) |
|  |  |  | I. longimana | (Machado, Teixeira, et al., 2019) | (Machado, Teixeira, et al., 2019) |
|  |  |  | I. similis | (Machado, Teixeira, et al., 2019) | (Machado, Teixeira, et al., 2019) |
|  |  |  | I. spiralis | (Machado, Teixeira, et al., 2019) | (Machado, Teixeira, et al., 2019) |
| Mecaphesa | EFS | (Hanna & Eason, 2013; Robakiewicz & Daigle, 2004) | M. aikoae | (Schenkel, 1965) | (Schenkel, 1965) |
|  |  |  | M. anguliventris | (Suman, 1971) | (Suman, 1971) |
|  |  |  | M. asperata | (Dondale & Redner, 1978) | (Dondale & Redner, 1978) |
|  |  |  | M. bubulcus | (Suman, 1971) | (Suman, 1971) |
|  |  |  | M. californica | (Schenkel, 1965) | (Schenkel, 1965) |
|  |  |  | M. carletonica | (Dondale & Redner, 1978) | (Dondale & Redner, 1978) |
|  |  |  | M. cavata | (Suman, 1971) | (Suman, 1971) |
|  |  |  | M. celer | (Dondale & Redner, 1978) | (Dondale & Redner, 1978) |
|  |  |  | M. coloradensis | (Gertsch, 1939) | (Gertsch, 1939) |
|  |  |  | M. decora | (Banks, 1898) | (Gertsch, 1939) |
|  |  |  | M. deserti | (Schenkel, 1965) | (Schenkel, 1965) |
|  |  |  | M. devia | (Gertsch, 1939) | (Gertsch, 1939) |
|  |  |  | M. discreta | (Suman, 1971) | (Suman, 1971) |
|  |  |  | M. dubia | (Gertsch, 1939) | (Gertsch, 1939) |
|  |  |  | M. edita | (Suman, 1971) | (Suman, 1971) |
|  |  |  | M. facunda | (Suman, 1971) | (Suman, 1971) |
|  |  |  | M. gabrielensis | (Schenkel, 1965) | (Schenkel, 1965) |
|  |  |  | M. imbricata | (Suman, 1971) | (Suman, 1971) |
|  |  |  | M. importuna | (Schenkel, 1965) | (Schenkel, 1965) |
|  |  |  | M. inclusa | (Baert, 2013) | (Baert, 2013) |
|  |  |  | M. insulana | (Keyserling, 1890) | (Keyserling, 1890) |
|  |  |  | M. juncta | (Suman, 1971) | (Suman, 1971) |
|  |  |  | M. lepida | (Schenkel, 1965) | (Schenkel, 1965) |
|  |  |  | M. lowriei | (Schick, 1970) | (Schick, 1970) |
|  |  |  | M. naevigera | (Suman, 1971) | (Suman, 1971) |
|  |  |  | M. nigrofrenata | (Suman, 1971) | (Suman, 1971) |
|  |  |  | M. oreades | (Suman, 1971) | (Suman, 1971) |
|  |  |  | M. perkinsi | (Suman, 1971) | (Suman, 1971) |
|  |  |  | M. quercina | (Schenkel, 1965) | (Schenkel, 1965) |
|  |  |  | M. reddelli | (Baert, 2013) | (Baert, 2013) |
|  |  |  | M. revillagigedoensis | (Jiménez, 1991) | (Jiménez, 1991) |
|  |  |  | M. rothi | (Schenkel, 1965) | (Schenkel, 1965) |
|  |  |  | M. rufithorax | (Suman, 1971) | (Suman, 1971) |
|  |  |  | M. schlingeri | (Schenkel, 1965) | (Schenkel, 1965) |
|  |  |  | M. semispinosa | (Suman, 1971) | (Suman, 1971) |
|  |  |  | M. sierrensis | (Dondale & Redner, 1978) | (Dondale & Redner, 1978) |
|  |  |  | M. velata | (Suman, 1971) | (Suman, 1971) |
|  |  |  | M. verityi | (Schenkel, 1965) | (Schenkel, 1965) |
| Metadiaea | EFS | (Vieira, 2015) | M. fidelis | (de Mello-Leitão, 1929) | (de Mello-Leitão, 1929) |
| Misumena | EFS | (Fritz & Morse, 1985; Morse, 1981) | M. nana | (de Lessert, 1933) | (de Lessert, 1933) |
|  |  |  | M. peninsulana | (Banks, 1898) | (Banks, 1898) |
|  |  |  | M. vatia | (Ono, 1988) | (Ono, 1988) |
| Misumenoides | EFS | (Dodson et al., 2013; Stellwag & Dodson, 2010; Vieira, 2015) | M. annulipes | (Gertsch, 1939) | (Gertsch, 1939) |
|  |  |  | M. athleticus | (Teixeira & Lise, 2012) | (Teixeira & Lise, 2012) |
|  |  |  | M. dasysternon | (de Mello-Leitão, 1943) | (de Mello-Leitão, 1943) |
|  |  |  | M. formosipes | (Dondale & Redner, 1978) | (Dondale & Redner, 1978) |
|  |  |  | M. magnus | (Keyserling, 1880) | (Keyserling, 1880) |
|  |  |  | M. parvus | (Keyserling, 1880) | (Keyserling, 1880) |
|  |  |  | M. quetzaltocatl | (Jiménez, 1992) | (Jiménez, 1992) |
|  |  |  | M. vazquezae | (Jiménez, 1986) | (Jiménez, 1986) |
|  |  |  | M. vigilans | (Pickard-Cambridge, 1900) | (Pickard-Cambridge, 1900) |
|  |  |  | M. vulneratus | (de Mello-Leitão, 1929) | (de Mello-Leitão, 1929) |
| Misumenops | EFS | (Dippenaar-Schoeman, 1983; Garb, 2007; Holmberg, 1876 *apud* Lehtinen & Marusik, 2008; Vieira, 2015) | M. bellulus | (Lehtinen & Marusik, 2008) | (Lehtinen & Marusik, 2008) |
|  |  |  | M. conspersus | (Keyserling, 1880) | (Keyserling, 1880) |
|  |  |  | M. croceus | (Keyserling, 1880) | (Keyserling, 1880) |
|  |  |  | M. guianensis | (Lehtinen & Marusik, 2008) | (Lehtinen & Marusik, 2008) |
|  |  |  | M. hunanensis | (Yin et al., 2012) | (Yin et al., 2012) |
|  |  |  | M. maculissparsus | (Lehtinen & Marusik, 2008) | (Lehtinen & Marusik, 2008) |
|  |  |  | M. melloleitaoi | (Garb, 2007) | (Garb, 2007) |
|  |  |  | M. ocellatus | (Tullgren, 1905) | (Tullgren, 1905) |
|  |  |  | M. pallens | (Rinaldi, 1983) | (Rinaldi, 1983) |
|  |  |  | M. pallidus | (Lehtinen & Marusik, 2008) | (Lehtinen & Marusik, 2008) |
|  |  |  | M. rapaensis | (Ledoux & Hallé, 1995) | (Ledoux & Hallé, 1995) |
|  |  |  | M. rubrodecoratus | (Dippenaar-Schoeman, 1983) | (Dippenaar-Schoeman, 1983) |
|  |  |  | M. schiapelliae | (de Mello-Leitão, 1944) | (de Mello-Leitão, 1944) |
|  |  |  | M. spinulosissimus | (Berland, 1936) | (Denis, 1941) |
|  |  |  | M. temibilis | (Lehtinen & Marusik, 2008) | (Lehtinen & Marusik, 2008) |
|  |  |  | M. temihana | (Garb, 2007) | (Garb, 2007) |
|  |  |  | M. variegatus | (Keyserling, 1880) | (Keyserling, 1880) |
| Monaeses | non-EFS | (Dippenaar-Schoeman, 1984) | M. aciculus | (Ono, 1985b) | (Ono, 1985b) |
|  |  |  | M. austrinus | (Dippenaar-Schoeman, 1984) | (Dippenaar-Schoeman, 1984) |
|  |  |  | M. caudatus | Tang, L. R. & Song, D. X. (1988a). | (Chen & Zhang, 1995) |
|  |  |  | M. fasciculiger | (Jézéquel, 1964) | (Jézéquel, 1964) |
|  |  |  | M. fuscus | (Dippenaar-Schoeman, 1984) | (Dippenaar-Schoeman, 1984) |
|  |  |  | M. gibbus | (Dippenaar-Schoeman, 1984) | (Dippenaar-Schoeman, 1984) |
|  |  |  | M. griseus | (Pavesi, 1897) | (Dippenaar-Schoeman, 1984) |
|  |  |  | M. habamatinikus | (Barrion & Litsinger, 1995) | (Barrion & Litsinger, 1995) |
|  |  |  | M. israeliensis | (Yuan et al., 2019) | (Yuan et al., 2019) |
|  |  |  | M. paradoxus | (Dippenaar-Schoeman, 1984) | (Dippenaar-Schoeman, 1984) |
|  |  |  | M. pustulosus | (Dippenaar-Schoeman, 1984) | (Dippenaar-Schoeman, 1984) |
|  |  |  | M. quadrituberculatus | (Dippenaar-Schoeman, 1984) | (Dippenaar-Schoeman, 1984) |
| Onocolus | non-EFS | (Vieira, 2015) | O. compactilis | (de Mello-Leitão, 1929) | (de Mello-Leitão, 1929) |
|  |  |  | O. echinatus | (de Mello-Leitão, 1929) | (de Mello-Leitão, 1929) |
|  |  |  | O. echinicaudus | (de Mello-Leitão, 1929) | (de Mello-Leitão, 1929) |
|  |  |  | O. garruchus | (Lise, 1979a) | (Lise, 1979a) |
|  |  |  | O. infelix | (de Mello-Leitão, 1941) | (B. A. M. Soares & Soares, 1946) |
|  |  |  | O. intermedius | (de Mello-Leitão, 1934) | (de Mello-Leitão, 1929) |
|  |  |  | O. mitralis | (Lise, 1979b) | (Lise, 1979b) |
|  |  |  | O. pentagonus | (de Mello-Leitão, 1929) | (de Mello-Leitão, 1929) |
|  |  |  | O. simoni | (de Mello-Leitão, 1929) | (de Mello-Leitão, 1929) |
| Oxytate | non-EFS | (Benjamin, 2001) | O. argenteooculata | (Simon, 1886a) | (de Lessert, 1919) |
|  |  |  | O. bhutanica | (G. Tang et al., 2008) | (G. Tang et al., 2008) |
|  |  |  | O. capitulata | (G. Tang & Li, 2010a) | (G. Tang & Li, 2010a) |
|  |  |  | O. forcipata | (Y. J. Zhang & Yin, 1998) | (Y. J. Zhang & Yin, 1998) |
|  |  |  | O. hoshizuna | (Ono, 1978) | (Ono, 1978) |
|  |  |  | O. leruthi | (Jézéquel, 1964) | (Jézéquel, 1964) |
|  |  |  | O. multa | (G. Tang & Li, 2010a) | (G. Tang & Li, 2010a) |
|  |  |  | O. palmata | (K. Liu et al., 2017) | (K. Liu et al., 2017) |
|  |  |  | O. parallela | (Paik, 1985b) | (Paik, 1985b) |
|  |  |  | O. ribes | (Jézéquel, 1966) | (Jézéquel, 1964) |
|  |  |  | O. striatipes | (Paik, 1985a) | (Paik, 1985a) |
|  |  |  | O. subvirens | (Benjamin, 2001) | (Benjamin, 2001) |
|  |  |  | O. taprobane | (Benjamin, 2001) | (Benjamin, 2001) |
| Ozyptila | non-EFS | (Deltshev et al., 2016; Gawryszewski et al., 2017; Mcheidze, 2014) | O. aculipalpa | (Danişman & Coşar, 2021) | (Danişman & Coşar, 2021) |
|  |  |  | O. americana | (Dondale & Redner, 1975) | (Dondale & Redner, 1975) |
|  |  |  | O. arctica | (Almquist, 2006) | (Almquist, 2006) |
|  |  |  | O. atomaria | (Almquist, 2006) | (Almquist, 2006) |
|  |  |  | O. balcanica | (Deltshev et al., 2016) | (Deltshev et al., 2016) |
|  |  |  | O. beaufortensis | (Dondale & Redner, 1975) | (Dondale & Redner, 1975) |
|  |  |  | O. bejarana | (Urones, 1998) | (Urones, 1998) |
|  |  |  | O. biprominula | (G. Tang & Li, 2010a) | (G. Tang & Li, 2010a) |
|  |  |  | O. brevipes | (Almquist, 2006) | (Almquist, 2006) |
|  |  |  | O. caenosa | (Jézéquel, 1964) | (Jézéquel, 1964) |
|  |  |  | O. claveata | (Almquist, 2006) | (Almquist, 2006) |
|  |  |  | O. clavigera | (Levy, 2007) | (Levy, 2007) |
|  |  |  | O. confluens | (Demircan & Topçu, 2015) | (Demircan & Topçu, 2015) |
|  |  |  | O. conspurcata | (Dondale & Redner, 1975) | (Dondale & Redner, 1975) |
|  |  |  | O. curvata | (Dondale & Redner, 1975) | (Dondale & Redner, 1975) |
|  |  |  | O. dagestana | (Ponomarev et al., 2011) | (Ponomarev et al., 2011) |
|  |  |  | O. distans | (Dondale & Redner, 1975) | (Dondale & Redner, 1975) |
|  |  |  | O. formosa | (Dondale & Redner, 1975) | (Dondale & Redner, 1975) |
|  |  |  | O. georgiana | (Dondale & Redner, 1975) | (Dondale & Redner, 1975) |
|  |  |  | O. gertschi | (Dondale & Redner, 1975) | (Dondale & Redner, 1975) |
|  |  |  | O. geumoensis | (Seo & Sohn, 1997) | (Seo, 2015) |
|  |  |  | O. imbrex | (G. Tang & Li, 2010b) | (G. Tang & Li, 2010b) |
|  |  |  | O. inaequalis | (Song & Zhu, 1997) | (Song & Zhu, 1997) |
|  |  |  | O. judaea | (Levy, 1985) | (Levy, 1985) |
|  |  |  | O. kaszabi | (Marusik & Logunov, 2002) | (Marusik & Logunov, 2002) |
|  |  |  | O. khasi | (Sen et al., 2015) | (Tikader, 1961) |
|  |  |  | O. ladina | (Teixeira & Barros, 2015) | (Teixeira & Barros, 2015) |
|  |  |  | O. lugubris | (Marusik & Logunov, 1990) | (Marusik & Logunov, 1990) |
|  |  |  | O. matsumotoi | (Ono, 1988) | (Ono, 1988) |
|  |  |  | O. monroensis | (Dondale & Redner, 1975) | (Dondale & Redner, 1975) |
|  |  |  | O. nipponica | (Ono, 1985a) | (Ono, 1985a) |
|  |  |  | O. nongae | (Ono, 1996) | (Ono, 1996) |
|  |  |  | O. pacifica | (Dondale & Redner, 1975) | (Dondale & Redner, 1975) |
|  |  |  | O. patellibidens | (Levy, 1999) | (Levy, 1999) |
|  |  |  | O. pauxilla | (Simon, 1875) | (Simon, 1875) |
|  |  |  | O. praticola | (Dondale & Redner, 1975) | (Dondale & Redner, 1975) |
|  |  |  | O. pullata | (Bell & Merrett, 2000) | (Bell & Merrett, 2000) |
|  |  |  | O. rauda | (Simon, 1875) | (Simon, 1875) |
|  |  |  | O. rigida | (Levy, 1975) | (Levy, 1975) |
|  |  |  | O. sakhalinensis | (Ono et al., 1990) | (Logunov & Marusik, 1994) |
|  |  |  | O. sanctuaria | (Simon, 1875) | (Simon, 1875) |
|  |  |  | O. scabricula | (Almquist, 2006) | (Almquist, 2006) |
|  |  |  | O. secreta | (Thaler, 1987) | (Thaler, 1987) |
|  |  |  | O. sedotmikha | (Levy, 2007) | (Levy, 2007) |
|  |  |  | O. simplex | (Roberts, 1998) | (Roberts, 1998) |
|  |  |  | O. sincera | (Ono, 1988) | (Ono, 1988) |
|  |  |  | O. tenerifensis | (Lissner, 2017) | (Lissner, 2017) |
|  |  |  | O. tricoloripes | (Levy, 1985) | (Levy, 1985) |
|  |  |  | O. trux | (Almquist, 2006) | (Almquist, 2006) |
|  |  |  | O. utotchkini | (Seo, 2015) | (Seo, 2015) |
|  |  |  | O. westringi | (Wunderlich & Schultz, 1995) | (Wunderlich & Schultz, 1995) |
|  |  |  | O. wuchangensis | (L. R. Tang & Song, 1988) | (L. R. Tang & Song, 1988) |
|  |  |  | O. yosemitica | (Dondale & Redner, 1975) | (Dondale & Redner, 1975) |
| Pagida | non-EFS | (Benjamin & Clayton, 2016) | P. minuta | (Benjamin & Clayton, 2016) | (Benjamin & Clayton, 2016) |
|  |  |  | P. pseudorchestes | (Benjamin & Clayton, 2016) | (Benjamin & Clayton, 2016) |
|  |  |  | P. salticiformis | (Benjamin & Clayton, 2016) | (Benjamin & Clayton, 2016) |
| Pharta | Unkown | - | P. bimaculata | (Ono, 1995) | (Ono, 1995) |
|  |  |  | P. brevipalpus | (Ono & Song, 1986) | (Ono & Song, 1986) |
|  |  |  | P. gongshan | (Yang et al., 2006) | (Yang et al., 2006) |
|  |  |  | P. sudmannorum | (Benjamin, 2014) | (Benjamin, 2014) |
|  |  |  | P. tangi | (C. Wang et al., 2016) | (C. Wang et al., 2016) |
| Phrynarachne | EFS | (Yu et al., 2022) | P. ceylonica | (Zhu & Song, 2006) | (Zhu & Song, 2006) |
|  |  |  | P. katoi | (Yin et al., 2012) | (S. T. Kim & Lee, 2012) |
|  |  |  | P. melloleitaoi | (de Lessert, 1933) | (de Lessert, 1933) |
|  |  |  | P. rugosa | (Ledoux, 2004) | (Ledoux, 2004) |
| Pistius | non-EFS | (Gawryszewski et al., 2017; S. T. Kim & Lee, 2012) | P. rotundus | (G. Tang & Li, 2010b) | (G. Tang & Li, 2010b) |
|  |  |  | P. truncatus | (Almquist, 2006) | (Almquist, 2006) |
|  |  |  | P. undulatus | (S. T. Kim & Lee, 2012) | (S. T. Kim & Lee, 2012) |
|  |  |  | P. wulingensis | (Tian et al., 2018) | (Tian et al., 2018) |
| Rejanellus | Unkown | - | R. mutchleri | (Lise, 2005) | (Lise, 2005) |
|  |  |  | R. pallescens | (Lise, 2005) | (Lise, 2005) |
|  |  |  | R. venustus | (Lise, 2005) | (Lise, 2005) |
| Runcinia | non-EFS | (Gawryszewski et al., 2017) | R. acuminata | (Ono, 1988) | (Ono, 1988) |
|  |  |  | R. aethiops | (Dippenaar-Schoeman, 1980) | (Dippenaar-Schoeman, 1980) |
|  |  |  | R. carae | (Dippenaar-Schoeman, 1983) | (Dippenaar-Schoeman, 1983) |
|  |  |  | R. depressa | (Dippenaar-Schoeman, 1980) | (Dippenaar-Schoeman, 1980) |
|  |  |  | R. erythrina | (Dippenaar-Schoeman, 1980) | (Dippenaar-Schoeman, 1980) |
|  |  |  | R. flavida | (Dippenaar-Schoeman, 1980) | (Dippenaar-Schoeman, 1980) |
|  |  |  | R. grammica | (Roberts, 1998) | (Roberts, 1998) |
|  |  |  | R. insecta | (Ono, 1988) | (Ono, 1988) |
|  |  |  | R. johnstoni | (Dippenaar-Schoeman, 1980) | (Dippenaar-Schoeman, 1980) |
|  |  |  | R. kinbergi | (Thorell, 1891) | (Thorell, 1895) |
|  |  |  | R. tropica | (de Lessert, 1919) | (de Lessert, 1919) |
| Sidymella | non-EFS | (Gawryszewski et al., 2017; Vieira, 2015) | S. excavata | (Machado, Guzati, et al., 2019) | (Machado, Guzati, et al., 2019) |
|  |  |  | S. furcillata | (Machado, Guzati, et al., 2019) | (Machado, Guzati, et al., 2019) |
|  |  |  | S. kolpogaster | (Machado, Guzati, et al., 2019) | (Machado, Guzati, et al., 2019) |
|  |  |  | S. longispina | (Machado, Guzati, et al., 2019) | (Machado, Guzati, et al., 2019) |
|  |  |  | S. lucida | (Machado, Guzati, et al., 2019) | (Machado, Guzati, et al., 2019) |
| Stephanopis | non-EFS | (Gawryszewski et al., 2017; Machado, Teixeira, et al., 2019) | S. altifrons | (Machado, Teixeira, et al., 2019) | (Machado, Teixeira, et al., 2019) |
|  |  |  | S. angulata | (Machado, Teixeira, et al., 2019) | (Machado, Teixeira, et al., 2019) |
|  |  |  | S. armata | (Machado, Teixeira, et al., 2019) | (Machado, Teixeira, et al., 2019) |
|  |  |  | S. barbipes | (Machado, Teixeira, et al., 2019) | (Machado, Teixeira, et al., 2019) |
|  |  |  | S. bicornis | (Machado, Teixeira, et al., 2019) | (Machado, Teixeira, et al., 2019) |
|  |  |  | S. carcinoides | (Machado, Teixeira, et al., 2019) | (Machado, Teixeira, et al., 2019) |
|  |  |  | S. fissifrons | (Machado, Teixeira, et al., 2019) | (Machado, Teixeira, et al., 2019) |
|  |  |  | S. lata | (Machado, Teixeira, et al., 2019) | (Machado, Teixeira, et al., 2019) |
|  |  |  | S. monulfi | (Machado, Teixeira, et al., 2019) | (Machado, Teixeira, et al., 2019) |
|  |  |  | S. nigra | (Machado, Teixeira, et al., 2019) | (Machado, Teixeira, et al., 2019) |
|  |  |  | S. squalida | (Machado, Teixeira, et al., 2019) | (Machado, Teixeira, et al., 2019) |
| Stephanopoides | non-EFS | (Vieira, 2015) | S. brasiliana | (Bonaldo & Lise, 2001) | (Bonaldo & Lise, 2001) |
|  |  |  | S. cognata | (Teixeira & Barros, 2015) | (Molina–Gómez et al., 2020) |
|  |  |  | S. sexmaculata | (Bonaldo & Lise, 2001) | (Bonaldo & Lise, 2001) |
|  |  |  | S. simoni | (Bonaldo & Lise, 2001) | (Bonaldo & Lise, 2001) |
| Stiphropus | non-EFS | (Z. Z. Yang et al., 2006) | S. affinis | (de Lessert, 1923) | (de Lessert, 1923) |
|  |  |  | S. qianlei | (Z.-X. Li et al., 2009) | (F. Li et al., 2023) |
|  |  |  | S. gruberi | (Ono, 1980) | (Ono, 1980) |
|  |  |  | S. melas | (Jézéquel, 1966) | (Jézéquel, 1966) |
|  |  |  | S. myrmecophilus | (Huang & Lin, 2020) | (Huang & Lin, 2020) |
|  |  |  | S. niger | (Millot, 1942) | (de Lessert, 1943) |
|  |  |  | S. ocellatus | (Zhu & Shan, 2007) | (Zhu & Shan, 2007) |
|  |  |  | S. scutatus | (Lawrence, 1928) | (Lawrence, 1927) |
|  |  |  | S. strandi | (Marusik & Logunov, 1995) | (Marusik & Logunov, 1995) |
| Strigoplus | Unkown | - | S. albostriatus | (Simon, 1885) | (Simon, 1885) |
|  |  |  | S. guizhouensis | (Yin et al., 2012) | (Yin et al., 2012) |
|  |  |  | S. netravati | (Dhali et al., 2016) | (Dhali et al., 2016) |
| Strophius | EFS | (Vieira, 2015) | S. albofasciatus | (de Mello-Leitão, 1929) | (de Mello-Leitão, 1929) |
|  |  |  | S. fidelis | (de Mello-Leitão, 1929) | (de Mello-Leitão, 1929) |
|  |  |  | S. mendax | (de Mello-Leitão, 1929) | (de Mello-Leitão, 1929) |
|  |  |  | S. nigricans | (de Mello-Leitão, 1929) | (de Mello-Leitão, 1929) |
| Synema | EFS | (Gawryszewski et al., 2017) | S. affinitatum | (Pickard-Cambridge, 1900) | (Pickard-Cambridge, 1900) |
|  |  |  | S. albomaculatum | (Ono, 2001) | (Ono, 2001) |
|  |  |  | S. anatolicum | (Demir et al., 2009) | (Demir et al., 2009) |
|  |  |  | S. bishopi | (di Caporiacco, 1955) | (di Caporiacco, 1955) |
|  |  |  | S. chikunii | (Ono, 1983) | (Ono, 1983) |
|  |  |  | S. diana | (Levy, 1975) | (Levy, 1975) |
|  |  |  | S. globosum |  |  |
|  |  |  | S. haenschi | (de Mello-Leitão, 1929) | (de Mello-Leitão, 1929) |
|  |  |  | S. imitatrix | (Pavesi, 1883) | (de Lessert, 1919) |
|  |  |  | S. lopezi | (Jiménez, 1988) | (Jiménez, 1988) |
|  |  |  | S. maculatovittatum | (di Caporiacco, 1954) | (di Caporiacco, 1954) |
|  |  |  | S. nigrianum | (B. M. Soares, 1943) | (B. A. M. Soares & Soares, 1946) |
|  |  |  | S. opulentum | (Thorell, 1895) | (Thorell, 1895) |
|  |  |  | S. parvulum | (Gertsch, 1939) | (Gertsch, 1939) |
|  |  |  | S. plorator | (Levy, 1975) | (Levy, 1975) |
|  |  |  | S. quadratum | (de Mello-Leitão, 1929) | (de Mello-Leitão, 1929) |
|  |  |  | S. reimoseri | (de Lessert, 1928) | (de Lessert, 1928) |
|  |  |  | S. revolutum | (G. Tang & Li, 2010b) | (G. Tang & Li, 2010b) |
|  |  |  | S. utotchkini | (Marusik & Logunov, 1995) | (Marusik & Logunov, 1995) |
|  |  |  | S. vachoni | (Jézéquel, 1964) | (Jézéquel, 1964) |
|  |  |  | S. viridans | (Gertsch, 1939) | (Gertsch, 1939) |
|  |  |  | S. viridisterne | (Jézéquel, 1966) | (Jézéquel, 1966) |
| Tharpyna | non-EFS | (Gawryszewski et al., 2017) | T. decorata | (Karsch, 1878) | (Karsch, 1878) |
|  |  |  | T. speciosa | (Rainbow, 1920) | (Rainbow, 1920) |
| Tharrhalea | EFS | (Gawryszewski et al., 2017) | T. evanida | (Szymkowiak, 2014) | (Szymkowiak, 2014) |
|  |  |  | T. fusca | (Thorell, 1877) | (Thorell, 1877) |
|  |  |  | T. multopunctata | (Szymkowiak, 2014) | (Szymkowiak, 2014) |
|  |  |  | T. pulleinei | (Szymkowiak, 2014) | (Szymkowiak, 2014) |
| Thomisus | EFS | (Gawryszewski et al., 2012, 2017; Heiling et al., 2003, 2005) | T. albertianus | (Strand, 1913) | (de Lessert, 1943) |
|  |  |  | T. albohirtus | (Dippenaar-Schoeman & van Harten, 2007) | (Dippenaar-Schoeman & van Harten, 2007) |
|  |  |  | T. amadelphus | (Simon, 1909) | (Simon, 1909) |
|  |  |  | T. arabicus | (Simon, 1882) | (Simon, 1882) |
|  |  |  | T. australis | (Dippenaar-Schoeman, 1983) | (Dippenaar-Schoeman, 1983) |
|  |  |  | T. bidentatus | (Levy, 1973) | (Levy, 1973) |
|  |  |  | T. blandus | (Dippenaar-Schoeman, 1983) | (Dippenaar-Schoeman, 1983) |
|  |  |  | T. callidus | (Workman, 1896) | (Simon, 1906) |
|  |  |  | T. castaneiceps | (Simon, 1909) | (Simon, 1909) |
|  |  |  | T. citrinellus | (Dippenaar-Schoeman, 1983) | (Dippenaar-Schoeman, 1983) |
|  |  |  | T. dalmasi | (Dippenaar-Schoeman, 1983) | (Dippenaar-Schoeman, 1983) |
|  |  |  | T. daradioides | (Dippenaar-Schoeman, 1983) | (Dippenaar-Schoeman, 1983) |
|  |  |  | T. destefanii | (di Caporiacco, 1941) | (di Caporiacco, 1941) |
|  |  |  | T. godavariae | (Reddy & Patel, 1991) | (Reddy & Patel, 1991) |
|  |  |  | T. granulatus | (Dippenaar-Schoeman, 1983) | (Dippenaar-Schoeman, 1983) |
|  |  |  | T. granulifrons | (Simon, 1906) | (Simon, 1906) |
|  |  |  | T. guadahyrensis | (Keyserling, 1880) | (Keyserling, 1880) |
|  |  |  | T. jocquei | (Dippenaar-Schoeman, 1988) | (Dippenaar-Schoeman, 1988) |
|  |  |  | T. kitamurai | (Ono, 1988) | (Ono, 1988) |
|  |  |  | T. labefactus | (Ono, 1988) | (Ono, 1988) |
|  |  |  | T. lamperti | (Strand, 1907) | (Strand, 1907) |
|  |  |  | T. nepenthiphilus | (Fage, 1930) | (Fage, 1930) |
|  |  |  | T. okinawensis | (Ono, 1988) | (Ono, 1988) |
|  |  |  | T. onustus | (Levy, 1973) | (Levy, 1973) |
|  |  |  | T. penicillatus | (Simon, 1909) | (Simon, 1909) |
|  |  |  | T. retirugus | (Simon, 1909) | (Simon, 1909) |
|  |  |  | T. rigoratus | (Simon, 1906) | (Simon, 1906) |
|  |  |  | T. scrupeus | (Dippenaar-Schoeman, 1983) | (Dippenaar-Schoeman, 1983) |
|  |  |  | T. socotrensis | (Dippenaar-Schoeman & van Harten, 2007) | (Dippenaar-Schoeman & van Harten, 2007) |
|  |  |  | T. spectabilis | Chrysanthus, 1964 | Chrysanthus, 1964 |
|  |  |  | T. spiculosus | (Dippenaar-Schoeman, 1983) | (Dippenaar-Schoeman, 1983) |
|  |  |  | T. stenningi | (Dippenaar-Schoeman, 1983) |  |
|  |  |  | T. stoliczkai | (Thorell, 1895) | (Thorell, 1887) |
|  |  |  | T. tetricus | (Simon, 1890) | (Simon, 1890) |
|  |  |  | T. unidentatus | (Dippenaar-Schoeman & van Harten, 2007) | (Dippenaar-Schoeman & van Harten, 2007) |
|  |  |  | T. wangi | (Yin et al., 2012) | (Yin et al., 2012) |
|  |  |  | T. yemensis | (Dippenaar-Schoeman & van Harten, 2007) | (Dippenaar-Schoeman & van Harten, 2007) |
|  |  |  | T. zhui | (L. R. Tang & Song, 1988) | (L. R. Tang & Song, 1988) |
|  |  |  | T. zyuzini | (Marusik & Logunov, 1990) | (Demir et al., 2008) |
| Tmarus | non-EFS | (Dippenaar-Schoeman, 1985; Gawryszewski et al., 2017; Ileperuma Arachchi & Benjamin, 2019; S. T. Kim & Lee, 2012) | T. africanus | (Dippenaar-Schoeman, 1985) | (Dippenaar-Schoeman, 1985) |
|  |  |  | T. albolineatus | (Keyserling, 1880) | (Garcia-Neto, 1991) |
|  |  |  | T. amazonicus | (de Mello-Leitão, 1929) | (de Mello-Leitão, 1929) |
|  |  |  | T. angulatus | (Dondale & Redner, 1978) | (Dondale & Redner, 1978) |
|  |  |  | T. byssinus | (G. Tang & Li, 2010b) | (G. Tang & Li, 2010b) |
|  |  |  | T. caeruleus | (de Mello-Leitão, 1929) | (Keyserling, 1880) |
|  |  |  | T. cameliformis | (Dippenaar-Schoeman, 1985) | (Dippenaar-Schoeman, 1985) |
|  |  |  | T. camellinus | (de Mello-Leitão, 1929) | (de Mello-Leitão, 1929) |
|  |  |  | T. cancellatus | (Comellini, 1955) | (Simon, 1907) |
|  |  |  | T. circinalis | (Song & Zhu, 1997) | (Song & Zhu, 1997) |
|  |  |  | T. comellinii | (Dippenaar-Schoeman, 1985) | (Dippenaar-Schoeman, 1985) |
|  |  |  | T. contortus | (Chickering, 1950) | (Chickering, 1950) |
|  |  |  | T. cretatus | (Chickering, 1965) | (Chickering, 1965) |
|  |  |  | T. curvus | (Chickering, 1950) | (Chickering, 1950) |
|  |  |  | T. digitiformis | (Yang et al., 2005) | (Yang et al., 2005) |
|  |  |  | T. ehecatltocatl | (Jiménez, 1992) | (Jiménez, 1992) |
|  |  |  | T. elongatus | (de Mello-Leitão, 1929) | (de Mello-Leitão, 1929) |
|  |  |  | T. farri | (Chickering, 1966) | (Chickering, 1966) |
|  |  |  | T. floridensis | (Gertsch, 1939) | (Keyserling, 1884) |
|  |  |  | T. foliatus | (Dippenaar-Schoeman, 1985) | (Dippenaar-Schoeman, 1985) |
|  |  |  | T. formosus | (de Mello-Leitão, 1929) | (de Mello-Leitão, 1929) |
|  |  |  | T. galapagosensis | (Baert, 2013) | (Baert, 2013) |
|  |  |  | T. hazevensis | (Levy, 1973) | (Levy, 2007) |
|  |  |  | T. histrix | (di Caporiacco, 1954) | (di Caporiacco, 1954) |
|  |  |  | T. hiyarensis | (Ileperuma Arachchi & Benjamin, 2019) | (Ileperuma Arachchi & Benjamin, 2019) |
|  |  |  | T. humphreyi | (Chickering, 1965) | (Chickering, 1965) |
|  |  |  | T. hystrix | (Ileperuma Arachchi & Benjamin, 2019) | (Ileperuma Arachchi & Benjamin, 2019) |
|  |  |  | T. ineptus | (Pickard-Cambridge, 1900) | (Chickering, 1950) |
|  |  |  | T. intentus | (Pickard-Cambridge, 1900) | (Pickard-Cambridge, 1900) |
|  |  |  | T. jelskii | (Keyserling, 1880) | (Taczanowski, 1872) |
|  |  |  | T. jocosus | (Pickard-Cambridge, 1900) | (Pickard-Cambridge, 1900) |
|  |  |  | T. karolae | (Jézéquel, 1964) | (Jézéquel, 1964) |
|  |  |  | T. koreanus | (Paik, 1973) | (Paik, 1973) |
|  |  |  | T. lanyu | (J. X. Zhang et al., 2006) | (J. X. Zhang et al., 2006) |
|  |  |  | T. lapadui | (Jézéquel, 1964) | (Jézéquel, 1964) |
|  |  |  | T. littoralis | (Keyserling, 1880) | (Keyserling, 1880) |
|  |  |  | T. longqicus | (Song & Zhu, 1997) | (Song & Zhu, 1997) |
|  |  |  | T. malleti | (de Lessert, 1919) | (de Lessert, 1919) |
|  |  |  | T. manojkaushalyai | (Ileperuma Arachchi & Benjamin, 2019) | (Ileperuma Arachchi & Benjamin, 2019) |
|  |  |  | T. menglae | (G. Tang & Li, 2010b) | (G. Tang & Li, 2010b) |
|  |  |  | T. minutus | (Gertsch, 1939) | (Gertsch, 1939) |
|  |  |  | T. morosus | (Chickering, 1950) | (Chickering, 1950) |
|  |  |  | T. mundulus | (Pickard-Cambridge, 1900) | (Chickering, 1950) |
|  |  |  | T. ningshaanensis | (X. G. Wang & Xi, 1998) | (X. G. Wang & Xi, 1998) |
|  |  |  | T. obesus | (de Mello-Leitão, 1929) | (di Caporiacco, 1954) |
|  |  |  | T. obsecus | (Chickering, 1965) | (Chickering, 1965) |
|  |  |  | T. orientalis | (Song & Zheng, 1981) | (Schenkel, 1963) |
|  |  |  | T. piger | (Ono, 1977) | (Ono, 1977) |
|  |  |  | T. piochardi | (Bayram et al., 2007) | (Bayram et al., 2007) |
|  |  |  | T. planetarius | (Dippenaar-Schoeman, 1985) | (Dippenaar-Schoeman, 1985) |
|  |  |  | T. planquettei | (Jézéquel, 1966) | (Jézéquel, 1966) |
|  |  |  | T. plurituberculatus | (de Mello-Leitão, 1929) | (de Mello-Leitão, 1929) |
|  |  |  | T. polyandrus | (de Mello-Leitão, 1929) | (de Mello-Leitão, 1929) |
|  |  |  | T. pugnax | (de Mello-Leitão, 1929) | (Garcia-Neto, 1986) |
|  |  |  | T. pulchripes | (Thorell, 1894) | (Thorell, 1894) |
|  |  |  | T. punctatissimus | (S. T. Kim & Lee, 2012) | (S. T. Kim & Lee, 2012) |
|  |  |  | T. qinlingensis | (Song & Zhu, 1997) | (Song & Zhu, 1997) |
|  |  |  | T. rimosus | (Yin et al., 2012) | (Yin et al., 2012) |
|  |  |  | T. rubromaculatus | (Gertsch, 1939) | (Gertsch, 1939) |
|  |  |  | T. salai | (Schenkel, 1965) | (Schenkel, 1965) |
|  |  |  | T. shimojanai | (Ono, 1997) | (Ono, 1997) |
|  |  |  | T. songi | (G. Tang & Li, 2010b) | (G. Tang & Li, 2010b) |
|  |  |  | T. srisailamensis | (Rao et al., 2006) | (Rao et al., 2006) |
|  |  |  | T. studiosus | (Pickard-Cambridge, 1900) | (Pickard-Cambridge, 1900) |
|  |  |  | T. taiwanus | (Han & Zhu, 2008) | (Han & Zhu, 2008) |
|  |  |  | T. tuberculitibiis | (di Caporiacco, 1940) | (di Caporiacco, 1940) |
|  |  |  | T. variatus | (Keyserling, 1891) | (Keyserling, 1891) |
|  |  |  | T. vertumus | (Chickering, 1966) | (Chickering, 1966) |
|  |  |  | T. viridomaculatus | (Ileperuma Arachchi & Benjamin, 2019) | (Ileperuma Arachchi & Benjamin, 2019) |
|  |  |  | T. yaginumai | (Ono, 1988) | (Ono, 1988) |
| Xysticus | non-EFS | (Gawryszewski et al., 2017) | X. ukrainicus | (Ovtsharenko, 1979) | (Mcheidze, 2014) |
|  |  |  | X. acerbus | (van Helsdingen, 1986) | (van Helsdingen, 1986) |
|  |  |  | X. acquiescens | (Dondale & Redner, 1978) | (Dondale & Redner, 1978) |
|  |  |  | X. albertensis | (Dondale, 2008) | (Dondale, 2008) |
|  |  |  | X. alboniger | (Dondale & Redner, 1978) | (Dondale & Redner, 1978) |
|  |  |  | X. alpicola | (Kulczyński, 1909) | (Kulczyński, 1882) |
|  |  |  | X. alpinus | (Ballarin et al., 2018) | (Ballarin et al., 2018) |
|  |  |  | X. alsus | (Song & Wang, 1994) | (Song & Wang, 1994) |
|  |  |  | X. ampullatus | (Dondale & Redner, 1978) | (Dondale & Redner, 1978) |
|  |  |  | X. apachecus | (Gertsch, 1939) | (Gertsch, 1939) |
|  |  |  | X. aprilinus | (Gertsch, 1939) | (Gertsch, 1939) |
|  |  |  | X. argenteus | (Jézéquel, 1966) | (Jézéquel, 1966) |
|  |  |  | X. atevs | (Ovtsharenko, 1979) | (Ovtsharenko, 1979) |
|  |  |  | X. atrimaculatus | (S. T. Kim & Lee, 2012) | (S. T. Kim & Lee, 2012) |
|  |  |  | X. auctificus | (Dondale & Redner, 1978) | (Dondale & Redner, 1978) |
|  |  |  | X. audax | (Jantscher, 2001) | (Jantscher, 2001) |
|  |  |  | X. austrosibiricus | (L. Liu et al., 2015) | (L. Liu et al., 2015) |
|  |  |  | X. bacurianensis | (Ovtsharenko, 1979) | (Ovtsharenko, 1979) |
|  |  |  | X. banksi | (Dondale & Redner, 1978) | (Dondale & Redner, 1978) |
|  |  |  | X. benefactor | (Dondale & Redner, 1978) | (Dondale & Redner, 1978) |
|  |  |  | X. bermani | (Guo et al., 2015) | (Guo et al., 2015) |
|  |  |  | X. bicuspis | (Dondale & Redner, 1978) | (Dondale & Redner, 1978) |
|  |  |  | X. bifasciatus | (Almquist, 2006) | (Almquist, 2006) |
|  |  |  | X. bimaculatus | (Ruch et al., 2014) | (Ruch et al., 2014) |
|  |  |  | X. bolivari | (Gertsch, 1953) | (Gertsch, 1953) |
|  |  |  | X. brevidentatus | (Bauer et al., 2020) | (Bauer et al., 2020) |
|  |  |  | X. britcheri | (Dondale & Redner, 1978) | (Dondale & Redner, 1978) |
|  |  |  | X. californicus | (Schenkel, 1965) | (Schenkel, 1965) |
|  |  |  | X. canadensis | (Dondale & Redner, 1978) | (Dondale & Redner, 1978) |
|  |  |  | X. chippewa | (Dondale et al., 2006) | (Dondale et al., 2006) |
|  |  |  | X. chui | (Song & Zhu, 1997) | (Song & Zhu, 1997) |
|  |  |  | X. concretus | (B. W. Kim & Lee, 2007) | (B. W. Kim & Lee, 2007) |
|  |  |  | X. concursus | (Gertsch, 1939) | (Gertsch, 1939) |
|  |  |  | X. conflatus | (Song & Zhu, 1997) | (Song & Zhu, 1997) |
|  |  |  | X. cristatus | (Almquist, 2006) | (Almquist, 2006) |
|  |  |  | X. croceus | (Yin et al., 2012) | (Yin et al., 2012) |
|  |  |  | X. cunctator | (Dondale & Redner, 1978) | (Dondale & Redner, 1978) |
|  |  |  | X. dali | (Z. X. Li & Yang, 2008) | (Z. X. Li & Yang, 2008) |
|  |  |  | X. desidiosus | (de Lessert, 1910) | (de Lessert, 1910) |
|  |  |  | X. discursans | (Dondale & Redner, 1978) | (Dondale & Redner, 1978) |
|  |  |  | X. dzhungaricus | (Marusik & Logunov, 1990) | (Marusik & Logunov, 1990) |
|  |  |  | X. edax | (Levy, 1976) | (Levy, 1976) |
|  |  |  | X. elegans | (Dondale & Redner, 1978) | (Dondale & Redner, 1978) |
|  |  |  | X. ellipticus | (Dondale & Redner, 1978) | (Dondale & Redner, 1978) |
|  |  |  | X. emertoni | (Dondale & Redner, 1978) | (Dondale & Redner, 1978) |
|  |  |  | X. ephippiatus | (Yin et al., 2012) | (Yin et al., 2012) |
|  |  |  | X. erraticus | (Almquist, 2006) | (Almquist, 2006) |
|  |  |  | X. ferox | (Dondale & Redner, 1978) | (Dondale & Redner, 1978) |
|  |  |  | X. ferrugineus | (Roberts, 1998) | (Roberts, 1998) |
|  |  |  | X. ferruginoides | (Schenkel, 1963) | (Schenkel, 1963) |
|  |  |  | X. fervidus | (Dondale & Redner, 1978) | (Dondale & Redner, 1978) |
|  |  |  | X. fraternus | (Dondale & Redner, 1978) | (Dondale & Redner, 1978) |
|  |  |  | X. funestus | (Dondale & Redner, 1978) | (Dondale & Redner, 1978) |
|  |  |  | X. furtivus | (Gertsch, 1939) | (Gertsch, 1939) |
|  |  |  | X. gallicus | (Ono & Martens, 2005) | (Ono & Martens, 2005) |
|  |  |  | X. gertschi | (Dondale & Redner, 1978) | (Dondale & Redner, 1978) |
|  |  |  | X. gosiutus | (Dondale & Redner, 1978) | (Dondale & Redner, 1978) |
|  |  |  | X. gracilis | (Keyserling, 1880) | (Keyserling, 1880) |
|  |  |  | X. grallator | (Carrillo et al., 2016) | (Carrillo et al., 2016) |
|  |  |  | X. guizhou | (Song & Zhu, 1997) | (Song & Zhu, 1997) |
|  |  |  | X. gulosus | (Dondale & Redner, 1978) | (Dondale & Redner, 1978) |
|  |  |  | X. hedini | (S. T. Kim & Lee, 2012) | (S. T. Kim & Lee, 2012) |
|  |  |  | X. humilis | (Redner & Dondale, 1965) | (Redner & Dondale, 1965) |
|  |  |  | X. insulicola | (Yin et al., 2012) | (Yin et al., 2012) |
|  |  |  | X. iviei | (Schenkel, 1965) | (Schenkel, 1965) |
|  |  |  | X. jiangi | (Yin et al., 2012) | (Yin et al., 2012) |
|  |  |  | X. kansuensis | (G. Tang et al., 2013) | (G. Tang et al., 2013) |
|  |  |  | X. kaznakovi | (Demir, 2015) | (Demir, 2015) |
|  |  |  | X. kempeleni | (Levy, 1976) | (Levy, 1976) |
|  |  |  | X. kochi | (Almquist, 2006) | (Almquist, 2006) |
|  |  |  | X. kulczynskii | (Logunov et al., 2002) | (Logunov et al., 2002) |
|  |  |  | X. kurilensis | (Yin et al., 2012) | (Yin et al., 2012) |
|  |  |  | X. laetus | (Logunov, 2006) | (Logunov, 2006) |
|  |  |  | X. lanio | (Almquist, 2006) | (Almquist, 2006) |
|  |  |  | X. lassanus | (Gertsch, 1939) | (Gertsch, 1939) |
|  |  |  | X. lepnevae | (B. W. Kim & Lee, 2007) | (B. W. Kim & Lee, 2007) |
|  |  |  | X. lineatus | (Almquist, 2006) | (Almquist, 2006) |
|  |  |  | X. locuples | (Dondale & Redner, 1978) | (Dondale & Redner, 1978) |
|  |  |  | X. logunovi | (Ono & Martens, 2005) | (Ono & Martens, 2005) |
|  |  |  | X. luctans | (Dondale & Redner, 1978) | (Dondale & Redner, 1978) |
|  |  |  | X. luctator | (Almquist, 2006) | (Almquist, 2006) |
|  |  |  | X. luctuosus | (van Helsdingen, 1986) | (van Helsdingen, 1986) |
|  |  |  | X. lutzi | (Gertsch, 1939) | (Gertsch, 1939) |
|  |  |  | X. macedonicus | (Jantscher, 2001) | (Jantscher, 2001) |
|  |  |  | X. maculiger | (di Caporiacco, 1935) | (di Caporiacco, 1935) |
|  |  |  | X. marmoratus | (Hepner et al., 2011) | (Hepner et al., 2011) |
|  |  |  | X. marusiki | (Ono & Martens, 2005) | (Ono & Martens, 2005) |
|  |  |  | X. mongolicus | (Utochkin & Savelyeva, 1995) | (Utochkin & Savelyeva, 1995) |
|  |  |  | X. montanensis | (Dondale & Redner, 1978) | (Dondale & Redner, 1978) |
|  |  |  | X. mugur | (Logunov & Marusik, 1994) | (Marusik & Chevrizov, 1990) |
|  |  |  | X. natalensis | (Lawrence, 1952) | (Lawrence, 1938) |
|  |  |  | X. nitidus | (J. Hu, 2001) | (J. Hu, 2001) |
|  |  |  | X. nubilus | (Thorell, 1875) | (Thorell, 1875) |
|  |  |  | X. obscurus | (Dondale & Redner, 1978) | (Dondale & Redner, 1978) |
|  |  |  | X. ocala | (Gertsch, 1953) | (Gertsch, 1953) |
|  |  |  | X. orizaba | (Gertsch, 1939) | (Gertsch, 1939) |
|  |  |  | X. ovatus | (Simon, 1876) | (Bosmans & Jacobs, 1985) |
|  |  |  | X. paiutus | (Gertsch, 1933) | (Gertsch, 1933) |
|  |  |  | X. palawanicus | (Barrion & Litsinger, 1995) | (Barrion & Litsinger, 1995) |
|  |  |  | X. parallelus | (Simon, 1873) | (Simon, 1873) |
|  |  |  | X. pellax | (Dondale & Redner, 1978) | (Dondale & Redner, 1978) |
|  |  |  | X. peninsulanus | (Gertsch, 1939) | (Gertsch, 1939) |
|  |  |  | X. pretiosus | (Dondale & Redner, 1978) | (Dondale & Redner, 1978) |
|  |  |  | X. promiscuus | (Levy, 1976) | (Levy, 1976) |
|  |  |  | X. pseudobliteus | (S. T. Kim & Lee, 2012) | (S. T. Kim & Lee, 2012) |
|  |  |  | X. pseudocristatus | (Azarkina & Logunov, 2001) | (Azarkina & Logunov, 2001) |
|  |  |  | X. pulcherrimus | (Keyserling, 1880) | (Keyserling, 1880) |
|  |  |  | X. punctatus | (Dondale & Redner, 1978) | (Dondale & Redner, 1978) |
|  |  |  | X. robinsoni | (Cockendolpher & Horner, 1980) | (Gertsch, 1953) |
|  |  |  | X. rockefelleri | (Gertsch, 1953) | (Gertsch, 1953) |
|  |  |  | X. rostratus | (Ono, 1988) | (Ono, 1988) |
|  |  |  | X. ryukyuensis | (Ono, 2002) | (Ono, 2002) |
|  |  |  | X. saganus | (S. T. Kim & Lee, 2012) | (S. T. Kim & Lee, 2012) |
|  |  |  | X. sagittifer | (Lawrence, 1927) | (Lawrence, 1928) |
|  |  |  | X. schoutedeni | (de Lessert, 1943) | (de Lessert, 1943) |
|  |  |  | X. sharlaa | (Zuo et al., 2014) | (Zuo et al., 2014) |
|  |  |  | X. siciliensis | IJland & van Helsdingen, 2016 | (Wunderlich, 1995) |
|  |  |  | X. sicus | (Di & Zhu, 2008) | (Di & Zhu, 2008) |
|  |  |  | X. sikkimus | (Ono, 1978) | (Tikader, 1980) |
|  |  |  | X. sjostedti | (Guo et al., 2015) | (Guo et al., 2015) |
|  |  |  | X. slovacus | (Svatoň et al., 2000) | (Svatoň et al., 2000) |
|  |  |  | X. soldatovi | (Song & Zhu, 1997) | (Song & Zhu, 1997) |
|  |  |  | X. spasskyi | (Marusik & Azarkina, 2016) | (Marusik & Azarkina, 2016) |
|  |  |  | X. tenuiapicalis | (Demir & Koçyiğit, 2016) | (Demır, 2012) |
|  |  |  | X. texanus | (Gertsch, 1939) | (Gertsch, 1939) |
|  |  |  | X. thessalicoides | (Wunderlich, 1995) | (Wunderlich, 1995) |
|  |  |  | X. thessalicus | (Karol, 1968) | (Karol, 1966) |
|  |  |  | X. tortuosus | (Simon, 1875) | (Simon, 1875) |
|  |  |  | X. transversomaculatus | (J. P. Kim et al., 2016) | (J. P. Kim et al., 2016) |
|  |  |  | X. triguttatus | (Dondale & Redner, 1978) | (Dondale & Redner, 1978) |
|  |  |  | X. trizonatus | (Ono, 1988) | (Ono, 1988) |
|  |  |  | X. tsanghoensis | (J. Hu, 2001) | (J. Hu, 2001) |
|  |  |  | X. turkmenicus | (Marusik & Logunov, 1995) | (Marusik & Logunov, 1995) |
|  |  |  | X. ulmi | (Almquist, 2006) | (Almquist, 2006) |
|  |  |  | X. urbensis | (Lawrence, 1952) | (Lawrence, 1952) |
|  |  |  | X. variabilis | (Gertsch, 1939) | (Gertsch, 1939) |
|  |  |  | X. verneaui | (Schmidt, 1968) | (Schmidt, 1968) |
|  |  |  | X. viduus | (Jantscher, 2001) | (Jantscher, 2001) |
|  |  |  | X. winnipegensis | (Redner & Dondale, 1980) | (Dondale & Redner, 1978) |
|  |  |  | X. wunderlichi | (Guo, Zhao, et al., 2015) | (Guo, Zhao, et al., 2015) |
|  |  |  | X. xerodermus | (Levy, 1976) | (Levy, 1976) |
| Zygometis | EFS | (Gawryszewski et al., 2017; Shield & Strudwick, 2000) | Z. xanthogaster | (Shield & Strudwick, 2000) | (Shield & Strudwick, 2000) |

**REFERENCES**

Almquist, S. (2006). Swedish Araneae, part. 2 families Dictynidae to Salticidae. *Insect Systematics & Evolution*, 299–299.

Azarkina, G. N., & Logunov, D. V. (2001). Separation and distribution of Xysticus cristatus (Clerck, 1758) and X. Audax (Schrank, 1803) in eastern Eurasia, with description of species from the mountains of Central Asia (Aranei: Thomisidae). *Arthropoda Selecta*, *9*(2), 133–150.

Baert, L. (2013). The Thomisidae and Philodromidae (Araneae) of the Galápagos Islands (Ecuador). *European Journal of Taxonomy*, *43*. https://doi.org/10.5852/ejt.2013.43

Ballarin, F., Pantini, P., & Gobbi, M. (2018). Elevation to species level and redescription of Xysticus alpinus Kulczyński, 1887 stat. N. (Araneae, Thomisidae). *Zootaxa*, *4500*(2), 271. https://doi.org/10.11646/zootaxa.4500.2.7

Banks, N. (1898). Arachnida from Baja California and other parts of Mexico. *Proceedings of the California Academy of Sciences*, *1*(3), 205–309.

Barrion, A. T., & Litsinger, J. A. (1995). *Riceland spiders of South and Southeast Asia* (C.A.B. International & International Rice Research Institute, Eds.). CAB International ; International Rice Research Institute.

Bauer, T., Lemke, M., & Pantini, P. (2020). Xysticus brevidentatus new to Bosnia and Herzegovina (Araneae: Thomisidae), with remarks on the habitat and distribution. *Arachnologische Mitteilungen: Arachnology Letters*, *60*(1), 34–37.

Bayram, A., Danışman, T., & Özgen. (2007). Two records new for the Turkish araneofauna: Tmarus piochardi (Simon, 1866) and Monaeses israeliensis Levy, 1973 (Araneae: Thomisidae). *Munis Entomology and Zoology*, *2*, 129–136.

Bell, D., & Merrett, P. (2000). Ozyptila pullata (Thorell, 1875), a thomisid spider new to Britain (Araneae: Thomisidae). *Bulletin of the British Arachnological Society*, *11*, 391–392.

Benjamin, S. P. (2000). Epidius parvati sp. n., a new species of the genus Epidius from Sri Lanka (Araneae: Thomisidae). *Bulletin-British Arachnological Society*, *11*(7), 284–288.

Benjamin, S. P. (2001). The genus Oxytate L. Koch 1878 from Sri Lanka, with description of Oxytate taprobane sp. n.(Araneae: Thomisidae). *Journal of South Asian Natural History*, *5*(2), 153–158.

Benjamin, S. P. (2011). Phylogenetics and comparative morphology of crab spiders (Araneae: Dionycha, Thomisidae). *Zootaxa*, *3080*, 1–108.

Benjamin, S. P. (2014). Two new species of Pharta Thorell, 1891 with the description of Ibana senagang gen. et sp. Nov. (Araneae: Thomisidae). *Zootaxa*, *3894*(1), 177. https://doi.org/10.11646/zootaxa.3894.1.15

Benjamin, S. P. (2015). On the African Crab Spider Genus Geraesta Simon, 1889 (Araneae: Thomisidae). *African Invertebrates*, *56*(2), 309–318. https://doi.org/10.5733/afin.056.0205

Benjamin, S. P. (2016). Revision Of Cebrenninus Simon, 1887 With Description Of One New Genus And Six New Species (Araneae: Thomisidae). *Revue Suisse de Zoologie*, *123*(1), 179–200. https://doi.org/10.5281/ZENODO.46304

Benjamin, S. P. (2017). Distributional and taxonomic notes on the crab spider genus *Epidius* with descriptions of five new species (Araneae: Thomisidae). *Journal of Natural History*, *51*(9–10), 469–485. https://doi.org/10.1080/00222933.2017.1302016

Benjamin, S. P., & Clayton, C. I. (2016). Phylogenetic placement and revision of the tropical Asian crab spider genus Pagida (Araneae: Thomisidae). *Invertebrate Systematics*, *30*(4), 353–369. https://doi.org/10.1071/IS15054

Berland, L. (1936). Mission de M. A. Chevalier aux iles du Cap Vert (1934). 1. Araignées. *Revue Française d’Entomologie*, *3*, 67–88.

Bonaldo, A. B., & Lise, A. A. (2001). A review of the Neotropical spider genus Stephanopoides (Araneae, Thomisidae, Stephanopinae). *Biociências*, *9*(1), 63–80.

Bosmans, R., & Jacobs, J. (1985). Description of the unknown male of Xysticus ovatus Simon from the French Pyrénées (Araneae: Thomisidae). *Phegea*, *13*.

Bryant, E. B. (1933). *Notes on types of Urquhart’s spiders*.

Bryant, E. B. (1935). Some new and little known species of New Zealand spiders. *Records of the Canterbury Museum*, *4*, 71–94.

Buchar, J., & Thaler, K. (1984). Eine zweite diaea-art in Mitteleuropa: Diaea pictilis (Araneida, Thomisidae). *Vestnik Ceskoslovenske Spolecnosti Zoologicke*, *48*, 1–8.

Canals, J. (1933). *Arañas argentinas de la familia" Aphantochilidae"*. Canals.

Carrillo, J., da Silva, R., & Fernández, M. (2016). Nuevas citas de Xysticus grallator Simon, 1932 (Araneae: Thomisidae) de la península ibérica. *Revista Ibérica de Aracnología*, *28*, 129–133.

Chen, Z., & Zhang, Z. (1995). A description of the male spider of Monaeses caudatus (Araneae: Thomisidae). *Zhuxing Xuebao*, *4*(2), 140–141.

Chickering, A. M. (1950). The Spider Genus Tmarus (Thomisidae) in Panama. *Bulletin of the Museum of Comparative Zoology*, *103*, 213–255.

Chickering, A. M. (1965). Panamanian spiders of the genus Tmarus (Araneae, Thomisidae). *Bulletin of the Museum of Comparative Zoology*, *133*(7), 33.

Chickering, A. M. (1966). Five new species of the genus Tmarus (Araneae, Thomisidae) from the West Indies. *Psyche: A Journal of Entomology*, *72*(3), 229–240. https://doi.org/10.1155/1965/849804

Chrysanthus, F. (1964). Spiders from south New Guinea VI. *Nova Guinea, Zoology*, *28*, 87–104.

Chua, T. J. L., & Lim, M. L. M. (2012). Cross-habitat predation in Nepenthes gracilis: The red crab spider Misumenops nepenthicola influences abundance of pitcher dipteran larvae. *Journal of Tropical Ecology*, *28*(1), 97–104.

Cockendolpher, J. C., & Horner, N. V. (1980). The female of Xysticus robinsoni (Araneae: Thomisidae). *The Southwestern Naturalist*, *25*(1), 109–111.

Comellini, A. (1955). Notes sur les Thomisidae d’Afrique. *Revue de Zoologie et de Botanique Africaines*, *51*.

Cushing, P. E. (2012). Spider-Ant Associations: An Updated Review of Myrmecomorphy, Myrmecophily, and Myrmecophagy in Spiders. *Psyche: A Journal of Entomology*, *2012*, e151989. https://doi.org/10.1155/2012/151989

Danişman, T., & Coşar, İ. (2021). Redescription of Ozyptila aculipalpa Wunderlich, 1995 (Araneae: Thomisidae), with a Description of Unknown Female. *Entomological News*, *129*(4). https://doi.org/10.3157/021.129.0403

de Lessert, R. (1910). Catalogue des invertebres de la Suisse. Fasc. 3, Araignées. *Musée d’histoire Naturelle de Genève*, 635.

de Lessert, R. (1919). Araignées du Kilimandjaro et du Merou (suite). III. Thomisidae. *Revue Suisse de Zoologie*, *27*, 99–234.

de Lessert, R. (1923). Araignées du sud de l’Afrique. *Revue Suisse de Zoologie*, *30*, 161–212.

de Lessert, R. (1928). Araignees du Congo. Recueillies au cours de l’expedition organisee par l’American Museum (1909-1915). Deuxieme partie. *Revue Suisse de Zoologie*, *35*, 103–159.

de Lessert, R. (1930). Araignees du Congo. Recueillies au cours de l’expedition organisee par l’American Museum (1909-1915). *Troisi Eme Partie. Rev. Suisse Zool.*, *36*, 103–159.

de Lessert, R. (1933). Araignées d’Angola. Résultats de la Mission scientifique suisse en Angola 1928-1929. *Revue Suisse de Zoologie*, *40*(4), 85–159. https://doi.org/10.5962/bhl.part.117656

de Lessert, R. (1943). Araignées du Congo Belg (III). *Revue Suisse de Zoologie*, *50*, 305–338.

de Mello-Leitão, C. F. (1929). Aphantochilidas e thomisidas do Brasil. *Arquivos Do Museu Nacional Do Rio de Janeiro*, *31*, 9–359.

de Mello-Leitão, C. F. (1934). A new Brasilian Onocolus. *Revista Chilena de Historia Natural*, *1934*, 38, 48.

de Mello-Leitão, C. F. (1941). Aranhas do Paraná. *Arquivos Do Instituto Biológico, Sao Paolo*, *1*, 235–257.

de Mello-Leitão, C. F. (1943). Aranhas do Chile coligidas pelo Dr. JC Carvalho. *Revista Brasileira de Biologia*, *3*, 403–409.

de Mello-Leitão, C. F. (1944). Arañas de la provincia de Buenos Aires. *Revista Del Museo de La Plata (N.S., Zool.)*, *3*, 311–393.

de Mello-Leitão, C. F. (1951). Arañas de Maullin, colectadas por el ingeniero Rafael Barros V. *Revista Chilena de Historia Natural*, *51–53*, 327–338.

Deltshev, C., Blagoev, G., Komnenov, M., & Lazarov, S. (2016). Description of Ozyptila balcanica sp. N. From the Balkan Peninsula and its Comparison with the closely related O. Umbraculorum Simon, 1932 (Araneae: Thomisidae). *Acta Zoologica Bulgarica*, *68*(4), 483–490.

Demir, H. (2015). Redescription of Xysticus kaznakovi Utochkin, 1968 (Araneae: Thomisidae), with a Description of Unknown Female. *Acta Zoologica Bulgarica*, *67*(4), 461–464.

Demir, H., Aktaş, M., & Topçu, A. (2008). Two thomisid species new to the Turkish spider fauna: Ozyptila tricoloripes Strand, 1913 and Thomisus zyuzini Marusik & Logunov, 1990 (Araneae: Thomisidae). *Turkish Journal of Arachnology*, *1*(1).

Demir, H., Aktaş, M., & Topçu, A. (2009). A new species of the genus Synema Simon, 1864 (Araneae: Thomisidae) from Turkey. *Biologia (Lahore, Pakistan)*, *64*(4), 742–744. https://doi.org/10.2478/s11756-009-0128-1

Demir, H., & Koçyiğit, H. O. (2016). Description of the hitherto unknown female of Xysticus tenuiapicalis Demir, 2012 (Araneae: Thomisidae). *Zoology in the Middle East*, *62*(3), 277–278. https://doi.org/10.1080/09397140.2016.1226549

Demircan, N., & Topçu, A. (2015). A contribution to the spider fauna of the European part of Turkey (Araneae). *Serket*, *14*(4), 8.

Demır, H. (2012). Xysticus tenuiapicalis sp. Nov. (Araneae: Thomisidae) from Turkey. *Florida Entomologist*, *95*(2), 359–361. https://doi.org/10.1653/024.095.0218

Denis, J. (1941). Les araignées des îles Canaries. *Annales de La Société Entomologique de France*, *110*, 105–130.

Dhali, D. C., Raychaudhuri, D., & Sureshan, P. M. (2016). Description of the unknown male of Strigoplus netravati Tikader, 1963 (Araneae: Thomisidae), with a key to the Indian species of the genus. *Arachnida – Rivista Aracnologica Italiana*, *10*, 26–33.

di Caporiacco, L. (1935). *Aracnidi dell’Himalaia e del Karakoram raccolti dalla Missione Italiana al Karakoram (1929-VII)*. *13*, 113–250.

di Caporiacco, L. (1940). Aracnidi raccolte nella Reg. Dei Laghi Etiopici della Fossa Galla. *Atti Della Reale Accademia d’Italia*, *11*, 767–873.

di Caporiacco, L. (1941). Arachnida (esc. Acarina). Araneae. *Missione Biologica Sagan-Omo, Reale Accademia d’Italia, Roma*, *12*(Zoologia 6), 46–175.

di Caporiacco, L. (1954). Araignées de la Guyane Française du Muséum d’Histoire Naturelle de Paris. *Commentationes Pontificia Academia Scientiarum*, *16*, 45–193.

di Caporiacco, L. (1955). Estudios sobre los aracnidos de Venezuela. 2a parte: Araneae. *Acta Biologica Venezuelica*, *1*(16), 265–448.

Di, Z. Y., & Zhu, M. S. (2008). The new discovery of the male Xysticus sicus from China (Araneae: Thomisidae). *Acta Arachnologica Sinica*, *17*, 16–18.

Dippenaar-Schoeman, A. S. (1980). The crab-spiders of southern Africa (Araneae: Thomisidae). 1. The genus Runcinia Simon, 1875. *Journal of the Entomological Society of South Africa*, *43*, 22.

Dippenaar-Schoeman, A. S. (1983). *The spider genera Misumena, Misumenops, Runcinia and Thomisus (Araneae: Thomisidae) of southern Africa*.

Dippenaar-Schoeman, A. S. (1984). The crab-spiders of southern Africa (Araneae: Thomisidae). 4. The genus Monaeses Thorell. *Phytophylactica*, *16*(2), 101–116.

Dippenaar-Schoeman, A. S. (1985). The crab-spiders of Southern Africa (Aranea: Thomisidae). 5. The genus Tmarus Simon, 1875. *Phytophylactica*, *17*(3), 115–128.

Dippenaar-Schoeman, A. S. (1988). An annotated checklist of the crab-spiders of Malawi (araneae: Thomisidae). I: The genera Misumenops, Runcinia and Thomisus, with a description of a new species. *Revue de Zoologie Africaine (1974)*, *102*(4), 429–438.

Dippenaar-Schoeman, A. S., & van Harten, A. (2007). Crab spiders (Araneae: Thomisidae) from mainland Yemen and the Socotra Archipelago: Part 1. The genus Thomisus Walckenaer, 1805. *Fauna of Arabia*, *23*, 169–188.

Dodson, G. N., Lang, P. L., Jones, R. N., & Versprille, A. N. (2013). Specificity of attraction to floral chemistry in Misumenoides formosipes crab spiders. *The Journal of Arachnology*, *41*(1), 36–42. https://doi.org/10.1636/Hi11-94.1

Dondale, C. D. (2008). A new species of Xysticus (Araneae, Thomisidae) from Alberta, Canada. *The Journal of Arachnology*, *36*(3), 601–603.

Dondale, C. D., Kronestedt, T., & Buckle, D. J. (2006). Confirmation of the presence of Xysticus chippewa in Europe (Araneae, Thomisidae). *Bulletin-British Arachnological Society*, *13*(9), 361.

Dondale, C. D., & Redner, J. H. (1975). The genus Ozyptila in North America (Araneida, Thomisidae). *Journal of Arachnology*, *2*, 129–181.

Dondale, C. D., & Redner, J. H. (1978). *The insects and arachnids of Canada. Part 5. The crab spiders of Canada and Alaska. Araneae: Philodromidae and Thomisidae.* Minister of Supply and Services.

Esyunin, S. L., Tuneva, T. K., & Farzalieva, G. S. (2007). Remarks on the Ural spider fauna (Arachnida, Aranei), 12. Spiders of the steppe zone of Orenburg Region. *Arthropoda Selecta*, *16*, 43–63.

Fage, L. (1928). Araneae. In: Notes on the fauna of pitcher plants. *Journal of the Malaysian Branch of the Royal Asiatic Society*, *6*(3), 13–19.

Fage, L. (1930). Au sujet de deux araignées nouvelles trouvees dans les urnes de Nepenthes. *Treubia*, *12*, 23–28.

Fritz, R. S., & Morse, D. H. (1985). Reproductive success and foraging of the crab spider Misumena vatia. *Oecologia*, *65*(2), 194–200. https://doi.org/10.1007/BF00379217

Garb, J. E. (2007). A new species of thomisid spider (Araneae, Thomisidae) from the Society Islands with a description of the male of Misumenops melloleitaoi. *Journal of Arachnology*, *34*(2), 357–367. https://doi.org/10.1636/H05-81.1

Garcia-Neto, L. N. (1986). Descrição do macho de Tmarus pugnax Mello-Leitão, 1929 (Araneae-Thomisidae-Misumeninae). *Publicações Avulsas do Museu Nacional, Rio de Janeiro*, *66*, 39–42.

Garcia-Neto, L. N. (1991). Descrição do macho de Tmarus albolineatus Keyserling, 1880 (Araneae, Thomisidae). *Boletim Do Museu Nacional Do Rio de Janeiro (n. Ser., Zool.)*, *345*, 1–6.

Gawryszewski, F. M., Calero-Torralbo, M. A., Gillespie, R. G., Rodríguez-Gironés, M. A., & Herberstein, M. E. (2017). Correlated evolution between coloration and ambush site in predators with visual prey lures. *Evolution; International Journal of Organic Evolution*, *71*(8), 2010–2021. https://doi.org/10.1111/evo.13271

Gawryszewski, F. M., Llandres, A. L., & Herberstein, M. E. (2012). Relationship between colouration and body condition in a crab spider that lures pollinators. *Journal of Experimental Biology*, *215*(7), 1128–1136. https://doi.org/10.1242/jeb.060558

Gertsch, W. J. (1933). *Notes on American spiders of the family Thomisidae*. American Museum of Natural History.

Gertsch, W. J. (1939). A revision of the typical crab-spiders (Misumeninae) of America north of Mexico. *Bulletin of the American Museum of Natural History*, *76*.

Gertsch, W. J. (1953). The spider genera Xysticus, Coriarachne, and Oxyptila (Thomisidae, Misumeninae) in North America. *Bulletin of the American Museum of Natural History*, *102*, 415–482.

Guo, C.-H., Ren, Z. X., & Zhang, F. (2015). Two newly recorded species of the genus Xysticus from Bashang Plateau, Hebei of China (Araneae: Thomisidae). *Journal of Tianjin Normal University (Natural Science Edition)*, *35*(3), 44–49.

Guo, C.-H., & Zhang, F. (2014). First description of the male of Diaea mikhailovi (Araneae: Thomisidae). *Zootaxa*, *3815*(3), 447. https://doi.org/10.11646/zootaxa.3815.3.11

Guo, C.-H., Zhao, M., & Zhang, F. (2015). One newly recorded Xysticus species from Taihang Mountains, China (Araneae: Thomisidae). *Acta Arachnologica Sinica*, *24*(2), 76–79.

Han, G., & Zhu, M. (2008). A New Species of the Genus Tmarus and Discovery of the Male Tmarus taiwanus (Araneae: Thomisidae) from China. *Entomological News*, *119*(5), 459–463. https://doi.org/10.3157/0013-872X-119.5.459

Hanna, C. J., & Eason, P. K. (2013). Juvenile crab spiders (Mecaphesa asperata) use indirect cues to choose foraging sites. *Ethology Ecology & Evolution*, *25*(2), 161–173. https://doi.org/10.1080/03949370.2012.742464

Heiling, A. M., Cheng, K., Chittka, L., Goeth, A., & Herberstein, M. E. (2005). The role of UV in crab spider signals: Effects on perception by prey and predators. *Journal of Experimental Biology*, *208*(20), 3925–3931. https://doi.org/10.1242/jeb.01861

Heiling, A. M., Herberstein, M. E., & Chittka, L. (2003). Crab-spiders manipulate flower signals. *Nature*, *421*(6921), 334–334. https://doi.org/10.1038/421334a

Hepner, M., Milasowszky, N., Sigmund, E., & Waitzbauer, W. (2011). Die Spinnenfauna (Arachnida: Araneae) stillgelegter Abbauflächen in einem Steinbruch in Bad Deutsch-Altenburg (Österreich: Niederösterreich). *Arachnologische Mitteilungen*, *42*, 29–47. https://doi.org/10.5431/aramit4207

Holmberg, E. L. (1876). Arácnidos argentinos. *Anales de Agricultura de La República Argentina*, *4*, 1–30.

Hu, J. (2001). *Spiders in Qinghai-Tibet Plateau of China*. Henan Science and Technology Publishing House.

Hu, J. L., & Wu, W. G. (1989). *Spiders from agricultural regions of Xinjiang Uygur Autonomous Region, China*. Shandong University Publishing House.

Huang, GuiQiang., & Lin, YeJie. (2020). A new species of Stiphropus (Aranei: Thomisidae) from China and first documentation of myrmecophily in this genus. *Arthropoda Selecta*, *29*(1), 257–261. https://doi.org/10.15298/arthsel.29.2.12

IJLand, S., & van Helsdingen, PeterJ. (2016). On some spiders (Arachnida, Araneae) of Basilicata and Calabria, Italy. *Nieuwsbrief Spined*, *36*, 25–44.

Ileperuma Arachchi, I. S., & Benjamin, S. P. (2019). Twigs that are not twigs: Phylogenetic placement of crab spiders of the genus Tmarus of Sri Lanka with comments on the higher-level phylogeny of Thomisidae. *Invertebrate Systematics*, *33*(3), 575–595. https://doi.org/10.1071/IS18074

Jantscher, E. (2001). *Revision der Krabbenspinnengattung Xysticus CL Koch, 1835 (Araneae, Thomisidae) in Zentraleuropa* [Doktorwürde]. Naturwissenschaftlichen Fakultät der Karl-Franzens-Universität Graz.

Jézéquel, J. F. (1964). Araignées de la savane de Singrobo (Côte d’Ivoire). III.-Thomisidae. *Bulletin de l’Institut Français d’Afrique Noire, Sér. A*, *26*, 1103–1143.

Jézéquel, J. F. (1966). Araignées de la savane de Singrobo (Côte d’Ivoire). V.-Note complémentaire sur les Thomisidae. *Bulletin Du Museum D’Histoire Naturelle*, 613–630.

Jiménez, M.-L. (1986). Descripción de una nueva especie de la familia Thomisidae (Arachnida: Araneae) de México. *Anales Del Instituto de Biología, Universidad Nacional Autónoma de México, Serie Zoología*, *56*, Article 1.

Jiménez, M.-L. (1988). Dos nuevas arañas cangrejo (Araneae, Thomisidae) de México. *Journal of Arachnology*, *15*, 395–399.

Jiménez, M.-L. (1991). Araneofauna de las islas Revillagigedo, México. *Anales Del Instituto Biológico de La Universidad Nacional Autónoma de México (Zool.)*, *62*.

Jiménez, M.-L. (1992). New Species of Crab Spiders from Baja California Sur (Araneae: Thomisidae). *Journal of Arachnology*, 52–57.

Jocqué, R. (1993). A new species of *Coriarachne* from Spain (Araneae, Thomisidae). *Bulletin de l’Institut Royal Des Sciences Naturelles de Belgique (Ent.)*, *63*, 119–122.

Joseph, J., & Ambalaparambil, S. (2017). First record of Epidius parvati Benjamin, 2000 (Araneae: Thomisidae) from Pathiramanal Island, India. *Check List (Luís Felipe Toledo)*, *13*(3), 1–4.

Karol, S. (1966). Spiders of Ankara and environs with a description of a new species Xysticus turcicus (Araneae, Thomisidae). *Communications de La Faculté Des Sciences de l’Université d’Ankara, Serie C*, *11*, 15–32.

Karol, S. (1968). Description de deux espèces nouvelles de Thomisidae (Araneae) de Turquie. *Bulletin Du Muséum National d’Histoire Naturelle de Paris (2)*, *39*, 908–911.

Karsch, F. (1878). Exotisch-araneologisches. *Zeitschrift Für Die Gesammten Naturwissenschaften*, *51*, 323–333.

Karsch, F. (1880). Drei neue afrikanische Araneiden. *Mittheilungen Des Münchener Entomologischen Vereins*, *4*, 145–146.

Keyserling, E. G. (1880). *Die Spinnen Amerikas, I. Laterigradae*. Verlag von Bauer & Raspe (E. Kuster).

Keyserling, E. G. (1884). Neue Spinnen aus America. V. *erhandlungen der Kaiserlich-Königlichen Zoologisch-Botanischen Gesellschaft in Wien*, *33*, 649–684.

Keyserling, E. G. (1890). Die Arachniden Australiens, nach der Natur beschrieben und abgebildet. Zweiter Theil [Lieferung 37]. *Bauer & Raspe, Nürnberg*, 233–274.

Keyserling, E. G. (1891). *Die Spinnen Amerikas. Brasilianische Spinnen.* Bauer & Raspe, Nürnberg 3.

Kim, B. W., & Lee, W. (2007). Two poorly known species of the spider genus Xysticus (Arachnida: Araneae: Thomisidae) in Korea. *Integrative Biosciences*, *11*(2), 105–115.

Kim, J. P., Ye, S. H., & Kim, B. W. (2016). Redescription of Ozyptila scabricula (Westring, 1851) and two new record species of the genus Ozyptila, Xysticus (Araneae: Thomisidae) from Korea. *Korean Arachnology*, *32*(1), 7–18.

Kim, S. T., & Lee, S. Y. (2012). Arthropoda: Arachnida: Araneae: Thomisidae. Thomisid spiders. *Invertebrate Fauna of Korea*, *21*(9), 1–88.

Komnenov, M. (2017). New data on spider fauna (Araneae) of Shar Mountain, north-western Macedonia. *Proceedings of the 5th Congress of the Ecologists of Macedonia, with International Participation (Ohrid, 19th-22nd October 2016). Special Issues of the Macedonian Ecological Society*, *13*, 44–61.

Kritscher, E. (1962). *Ozyptila baudueri* Simon ssp. *cribratus* (Simon 1885) (=*Xysticus cribratus* Simon 1885) (Aran., Thomisidae). *Annalen des Naturhistorischen Museums in Wien*, *65*, 177–182.

Kulczyński, W. (1882). Opisy nowych Gatunkow Pająkow, z Tatra, Babiej gory i Karpat szlazkich przez. *Pamietnik Akademji Umiejetnosci w Krakow Wydzial Matematyczno-Przyrodniczy*, *8*, 1–42.

Kulczyński, W. (1901). *Arachnoidea in Colonia Erytraea a Dre KM Levander collecta*. Bibliopolam Societatis Librariae Polonicae.

Kulczyński, W. (1909). Fragmenta Arachnologica. XIV, XV. *Bulletin International de l’Académie Des Sciences de Cracovie*, *1909*, 667–687.

Kulczynski, W. (1911). Spinnen aus Süd-Neu-Guinea. In *Résultats de l’expédition scientifique néerlandaise a la Nouvelle Guinée en 1907 et 1909, sous les auspices du Dr H. A. Lorenz* (Vol. 9, pp. 109–148). Erster Teil.

Lawrence, R. F. (1927). Contributions to a knowledge of the fauna of South-West Africa. V. Arachnida. *Annals of the South African Museum*, *25*(1), 217–312.

Lawrence, R. F. (1928). Contributions to a knowledge of the fauna of South-West Africa. VII. Arachnida (Part 2). *Annals of the South African Museum*, *25*(1), 217–312.

Lawrence, R. F. (1938). *A collection of spiders from Natal and Zululand*.

Lawrence, R. F. (1952). New spiders from the eastern half of South Africa. *Annals of the Natal Museum*, *12*, 183–226.

Ledoux, J.-C. (2004). Araignées de l’île de La Réunion: I. Hahniidae, Ctenidae, Thomisidae et Clubionidae (Araneae). *Revue Arachnologique*, *14*(11), 159–191.

Ledoux, J.-C., & Hallé, N. (1995). Araignées de l’île Rapa (îles Australes, Polynésie). *Revue Arachnologique*, *11*(1), 1–15.

Lehtinen, P. T., & Marusik, Y. M. (2008). A redefinition of Misumenops F. O. Pickard-Cambridge, 1900 (Araneae, Thomisidae) and review of the New World species. *Bulletin of the British Arachnological Society*, *14*(4), 173–198.

Levy, G. (1973). Crab-spiders of six genera from Israel (Araneae: Thomisidae). *Israel Journal of Ecology and Evolution*, *22*(2–4), 107–141.

Levy, G. (1975). The spider genera Synaema and Oxyptila in Israel (Araneae: Thomisidae). *Israel Journal of Ecology and Evolution*, *24*(3–4), 155–175.

Levy, G. (1976). The spider genus Xysticus (Araneae: Thomisidae) in Israel. *Israel Journal of Ecology and Evolution*, *25*(1–2), 1–37.

Levy, G. (1985). Araneae: Thomisidae. In *Fauna Palaestina, Arachnida II* (p. 115). Israel Academy of Sciences and Humanities.

Levy, G. (1999). New thomisid and philodromid spiders (Araneae) from southern Israel. *Bulletin of the British Arachnological Society*, *11*(5), 185–190.

Levy, G. (2007). Calommata (Atypidae) and new spider species (Araneae) from Israel. *Zootaxa*, *1551*(1), 1–30. https://doi.org/10.11646/zootaxa.1551.1.1

Li, F., Lin, Y., & Li, S. (2023). Notes on two Stiphropus species from China (Araneae, Thomisidae). *Biodiversity Data Journal*, *11*, e105695. https://doi.org/10.3897/BDJ.11.e105695

Li, Z. X., & Yang, Z. Z. (2008). A new species of the genus Xysticus (Araneae: Thomisidae) from China. *Journal of Dali University*, *7*, 5–17.

Li, Z.-X., Zhou, Y.-F., & Yang, Z.-Z. (2009). First description of the female of Stiphropus falciformus (Araneae: Thomisidae). *Acta Arachnologica*, *58*(2), 65–66.

Liang, T., Zhu, C. D., & Wang, J. F. (1991). A new species of the genus Heriaeus from Xinjiang Uygur Autonomous Region. *Journal of August 1st Agricultural College*, *14*(3), 34–36.

Lise, A. A. (1979a). Tomisídeos neotropicais I: Onocolus garruchus Sp.n (Araneae—Thomisidae—Stephanopsinae). *Iheringia (Zool.)*, *54*, 67–76.

Lise, A. A. (1979b). Tomisídeos neotropicais, IV: Onocolus mitralis sp. N. (Araneae, Thomisidae, Stephanopsinae). *Revista Brasileira de Biologia*, *39*, 487–492.

Lise, A. A. (2005). *Rejanellus*, a new genus of Thomisidae (Araneae, Stephanopinae). *Iheringia. Série Zoologia*, *95*(2), 151–164. https://doi.org/10.1590/S0073-47212005000200005

Lissner, J. (2017). Description of the unknown male of *Ozyptila Tenerifensis* (araneae: Thomisidae). *Arachnologische Mitteilungen*, *53*, 50–52. https://doi.org/10.5431/aramit5308

Liu, K., Liu, J., & Xu, X. (2017). Two new species of the genus Oxytate from China (Araneae: Thomisidae). *Zootaxa*, *4320*(1), 193. https://doi.org/10.11646/zootaxa.4320.1.12

Liu, L., Guo, C., & Zhang, F. (2015). Two newly recorded species of the genus Xysticus (Araneae: Thomisidae) from China. *Journal of Hebei University (Natural Science Edition)*, *35*(1).

Loerbroks, A. (1983). Revision der Krabbenspinnen-Gattung Heriaeus Simon (Arachnida: Araneae: Thomisidae). *Verhandlungen Des Naturwissenschaftlichen Vereins in Hamburg (NF)*, *26*, 85–139.

Logunov, D. V. (2006). Notes on Xysticus kempeleni Thorell, 1872 and two closely related spider species (Araneae, Thomisidae). *Acta Arachnologica*, *55*(1), 59–66. https://doi.org/10.2476/asjaa.55.59

Logunov, D. V., & Marusik, Y. M. (1994). *A faunistic review of the crab spiders (Araneae, Thomisidae) from the mountains of South Siberia*. 177–197.

Logunov, D. V., Marusik, Y. M., & Koponen, S. (2002). Redescription of a poorly known spider species, Xysticus kulczynskii Wierzbicki 1902 (Araneae: Thomisidae). *Acta Arachnologica*, *51*(2), 99–104.

Machado, M., Guzati, C., Viecelli, R., Molina-Gómez, D., & Teixeira, R. A. (2019). A taxonomic review of the crab spider genus Sidymella (Araneae, Thomisidae) in the Neotropics. *Zoosystematics and Evolution*, *95*(2), 319–344. https://doi.org/10.3897/zse.95.34958

Machado, M., & Teixeira, R. A. (2021). Phylogenetic relationships in Stephanopinae: Systematics of Stephanopis and Sidymella based on morphological characters (Araneae: Thomisidae). *Organisms Diversity & Evolution*, *21*(2), 281–313. https://doi.org/10.1007/s13127-020-00472-x

Machado, M., Teixeira, R. A., & Lise, A. A. (2015). Taxonomic notes on the crab spider genus Tobias Simon, 1895 (Araneae, Thomisidae, Stephanopinae). *Zootaxa*, *4034*(3), 565. https://doi.org/10.11646/zootaxa.4034.3.8

Machado, M., Teixeira, R. A., & Lise, A. A. (2018). There and back again: More on the taxonomy of the crab spiders genus Epicadus (Thomisidae: Stephanopinae). *Zootaxa*, *4382*(3), 501. https://doi.org/10.11646/zootaxa.4382.3.4

Machado, M., Teixeira, R. A., & Milledge, G. A. (2019). On the Australian bark crab spiders genus Stephanopis: Taxonomic review and description of seven new species (Araneae: Thomisidae: Stephanopinae). *Records of the Australian Museum*, *71*(6), 217–276.

Marusik, Y. M. (1993). *Re-description of spiders of the families Heteropodidae and Thomisidae (Aranei), described by OP-Cambridge from the material of the second Yarkand mission.* *72*(2), 456–468.

Marusik, Y. M., & Azarkina, G. N. (2016). Redescription of the poorly known crab spider Xysticus spasskyi (Araneae: Thomisidae). *Zootaxa*, *4161*(4), 561. https://doi.org/10.11646/zootaxa.4161.4.7

Marusik, Y. M., & Chevrizov, B. P. (1990). Three new crab spiders from the Asian part of the USSR (Arachnida, Araneae: Thomisidae). *Reichenbachia*, *27*, 89–93.

Marusik, Y. M., & Logunov, D. V. (1990). The crab spiders of Middle Asia, USSR (Aranei, Thomisidae). 1. Descriptions and notes on distribution of some species. *Korean Arachnology*, *6*, 31–62.

Marusik, Y. M., & Logunov, D. V. (1995). *The crab spiders of Middle Asia (Aranei, Thomisidae), 2*. *4*, 133–176.

Marusik, Y. M., & Logunov, D. V. (2002). New and poorly known species of crab spiders (Aranei: Thomisidae) from South Siberia and Mongolia. *Arthropoda Selecta*, *10*(4, 2001), 315–322.

Mcheidze, T. (2014). *Georgian spiders: Systematics, ecology and zoogeographic analysis*. Universitätsbibliothek Johann Christian Senckenberg.

Meng, Z.-Y., Luo, H.-P., Xiao, Y.-H., Xu, X., & Liu, K.-K. (2019). Redescription of Borboropactus jiangyong Yin, Peng, Yan & Kim, 2004 (Araneae, Thomisidae), with the first description of the male. *ZooKeys*, *870*, 113–148. https://doi.org/10.3897/zookeys.870.35230

Millot, J. (1942). Les araignées de l’Afrique Occidentale Français: Thomisidae. *Mémoires de l’Académie Des Sciences de Paris*, *65*(2), 1–82.

Molina–Gómez, D., Pizzetti, D. dos P., & Teixeira, R. A. (2020). Taxonomic notes on Stephanopoides (Araneae: Thomisidae): New records and description of the male of S. Cognata. *Studies on Neotropical Fauna and Environment*, *55*(1), 51–57.

Morse, D. H. (1981). Prey Capture by the Crab Spider Misumena vatia (Clerck) (Thomisidae) on Three Common Native Flowers. *The American Midland Naturalist*, *105*(2), 358–367. https://doi.org/10.2307/2424754

Ono, H. (1977). Thomisidae aus Japan I. Das Genus Tmarus Simon (Arachnida: Araneae). *Acta Arachnologica*, *27*(Specialnumber), 61–84. https://doi.org/10.2476/asjaa.27.Specialnumber_61

Ono, H. (1978). *Thomisidae aus dem Nepal-Himalaya. I. Das Genus Xysticus C. L. Koch 1835 (Arachnida: Araneae).*

Ono, H. (1980). *Thomisidae aus dem Nepal-Himalaya. III. Das Genus Stiphropus Gerstaecker 1873, mit Revision der asiatischen Arten (Arachnida: Araneae).*

Ono, H. (1983). Eine neue japanische Synaema-Art (Araneae: Thomisidae). *Acta Arachnologica*, *31*(2), 59–63.

Ono, H. (1985a). Revision einiger arten der familie Thomisidae (Arachnida, Araneae) aus Japan. *Bulletin of the National Science Museum. Series A. Zoology*, *11*(1), 19–39.

Ono, H. (1985b). The Thomisidae of Japan. V: Monaeses Thorell, 1869, and its new junior synonym, Mecostrabus Simon, 1903 (Arachnida, Araneae). *Bulletin of the National Science Museum. Series A. Zoology*, *11*(2), 91–97.

Ono, H. (1988). *A revisional study of the spider family Thomisidae (Arachnida, Araneae) of Japan.* National Science Museum.

Ono, H. (1995). Four East Asian spiders of the families Eresidae, Araneidae, Thomisidae and Salticidae (Arachnida, Araneae). *Bulletin of the National Museum of Nature and Science, Tokyo (A)*, *21*, 157–169.

Ono, H. (1996). New records of two Korean species of the spider families Mimetidae and Thomisidae (Araneae) from Japan. *Acta Arachnologica*, *45*, 19–24.

Ono, H. (1997). New species of the genera Ryuthela and Tmarus (Araneae, Liphistiidae and Thomisidae) from the Ryukyu Islands, southwest Japan. *Bull. Nat. Sci. Mus., Tokyo,(A)*, *23*, 149–163.

Ono, H. (2001). Crab spiders of the family Thomisidae from the Kingdom of Bhutan (Arachnida, Araneae). *Entomologia Basiliensis*, *23*, 203–236.

Ono, H. (2002). New species of crab spiders (Araneae, Thomisidae) from Japan. *Bulletin of the National Museum of Nature and Science Tokyo (A)*, *28*, 201–210.

Ono, H., & Martens, J. (2005). Crab spiders of the families Thomisidae and Philodromidae (Arachnida: Araneae) from Iran. *Acta Arachnologica*, *53*(2), 109–124.

Ono, H., Marusik, Y. M., & Logunov, D. V. (1990). Spiders of the family Thomisidae from Sakhalin and the Kurile Islands. *Acta Arachnologica*, *39*, 7–19.

Ono, H., & Song, D. (1986). A new sino japanese species of the genus lupa (Araneae, Phomisidae) from the coastal areas of the East China sea. *Bulletin of the National Science Museum. Series A. Zoology*, *12*(1), 25–29.

Ovtsharenko, V. I. (1979). Spiders of the families Gnaphosidae, Thomisidae, Lycosidae (Aranei) of the Caucasus Major. *The Fauna and Ecology of Arachnida. Proc. Inst. Zool. Acad. Sci. URSS. Leningrad*, *85*, 28–38.

Paik, K. Y. (1973). Korean spiders of genus Tmarus (Araneae, Thomisidae). *Thes. Coll. Grad. Sch. Educ. Kyungpook Natn. Univ*, *4*, 79–89.

Paik, K. Y. (1985a). A new species of genus Oxyptila (Araneae: Thomisidae) from Korea. *Korean Arachnology*, *1*(1), 13–16.

Paik, K. Y. (1985b). Korean spiders of the genus Oxytate L. Koch, 1878 (Thomisidae: Araneae). *Korean Arachnology*, *1*(2), 29–42.

Pavesi, P. (1883). Studi sugli aracnidi africani. III. Aracnidi del regno di Scioa e considerazioni sull’aracnofauna d’Abissinia. *Annali Del Museo Civico Di Storia Naturale Di Genova*, *20*, 1–105.

Pavesi, P. (1897). Studi sugli aracnidi africani IX. Aracnidi Somali e Galla raccolti da Don Eugenio dei Principi Rispoli. *Annali Del Museo Civico Di Storia Naturale Di Genova*, *38*, 151–188.

Pickard-Cambridge, O. (1900). Arachnida-Araneida and Opiliones. *Biologia Centrali-Americana, Zoology*, *2*, 89–192.

Ponomarev, A. V., Abdurakhmanov, G. M., Alieva, S. V., & Dvadnenko, K. V. (2011). Spiders (Arachnida: Aranei) of coastal and island territories of northern Daghestan. *South of Russia: Ecology, Development*, *2011*(4), 126–143.

Prado, A. W. D., Baptista, R. L. C., & Machado, M. (2018). Taxonomic review of Epicadinus Simon, 1895 (Araneae: Thomisidae). *Zootaxa*, *4459*(2), 201. https://doi.org/10.11646/zootaxa.4459.2.1

Rainbow, W. J. (1920). Arachnida from Lord Howe and Norfolk Islands. *Records of the South Australian Museum*, *1*(3), 229–272.

Rao, K. T., Bastawade, D. B., Javed, S. M., & Krishna, I. S. R. (2006). Description of two new species of spiders of the genus Poecilotheria Simon (Araneae: Theraphosidae) and Tmarus Simon (Araneae: Thomisidae) from Nallamalai Hills, eastern Ghats, Andhra Pradesh, India. *Records of the Zoological Survey of India*, *106*(1), 49–54.

Reddy, T. S., & Patel, B. H. (1991). Two new species of the genus Thomisus Walckenaer (Araneae: Thomisidae) from coastal Andhra Pradesh. *Journal of the Bombay Natural History Society*, *88*(2), 268–272.

Redner, J. H., & Dondale, C. D. (1965). A New Species of The Spider Genus Xysticus (Araneae: Thomisidae) From Arizona. *Psyche*, *72*(4), 291–294.

Redner, J. H., & Dondale, C. D. (1980). Description of the female of Xysticus winnipegensis (Araneae: Thomisidae). *The Canadian Entomologist*, *112*(9), 933–934.

Rinaldi, I. M. P. (1983). Contribuição ao estudo das Misumeninae do Brasil (Araneae, Thomisidae). *Revista Brasileira de Entomologia*, *27*(2), 147–153.

Robakiewicz, P., & Daigle, W. (2004). Patch Quality and Foraging Time in the Crab Spider Misumenops Asperatus Hentz (Araneae: Thomisidae). *Northeastern Naturalist*, *11*(1), 23–32. https://doi.org/10.1656/1092-6194(2004)011[0023:PQAFTI]2.0.CO;2

Roberts, M. J. (1998). *Spinnengids*.

Ruch, J., Riehl, T., & Michalik, P. (2014). Re-description of Xysticus bimaculatus L. Koch, 1867 (Araneae, Thomisidae) and characterization of its subsocial lifestyle. *ZooKeys*, *427*, 1.

Schenkel, E. (1963). Ostasiatische Spinnen aus dem Muséum d’Histoire naturelle de Paris. *Mémoires Du Muséum National d’Histoire Naturelle de Paris (A, Zool.)*, *25*, 1-481.

Schick, R. X. (1965). The crab spiders of California (Araneida, Thomisidae). *Bulletin of the American Museum of Natural History*, *129*, 1–180.

Schick, R. X. (1970). A new thomisid from California. *Notes of the Arachnologists of the Southwest*, *1*.

Schmidt, G. (1968). Zur Spinnenfauna von Teneriffa. *Zoologische Beiträge (NF)*, *14*, 387–425.

Sen, S., Dhali, D. C., Saha, S., & Raychaudhuri, D. (2015). Spiders (Araneae: Arachnida) of Reserve Forests of Dooars: Gorumara National Park, Chapramari Wildlife Sanctuary and Mahananda Wildlife Sanctuary. *World Scientific News*, *20*, 339.

Seo, B. K. (2015). A new species of the spider genus Ebrechtella Dahl and first record of Ozyptila utotchkini Marusik, with the first description of the male of Ozyptila geumoensis Seo and Sohn (Araneae, Thomisidae) from Korea. *Korean Journal of Environmental Biology*, *33*(2), 112–118. https://doi.org/10.11626/KJEB.2015.33.2.112

Seo, B. K., & Sohn, S. R. (1997). A new species of genus Ozyptila (Araneae: Thomisidae) from Korea. *Journal of the Institute of Natural Sciences, Keimyung University*, *16*, 41–43.

Shield, J. M., & Strudwick, J. (2000). Diasterea, a new genus of flower spider (Thomisidae; Thomisinae) from eastern Australia and a description of the male Diasterea lactea. *Proceedings of the Royal Society of Victoria*, *111*, 271–281.

Silva-Moreira, T. D., & Machado, M. (2016). Taxonomic revision of the crab spider genus Epicadus Simon, 1895 (Arachnida: Araneae: Thomisidae) with notes on related genera of Stephanopinae Simon, 1895. *Zootaxa*, *4147*(3), 281. https://doi.org/10.11646/zootaxa.4147.3.4

Simon, E. (1873). Etudes arachnologiques. 2e Mémoire. II. Description de quelques espèces nouvelles pour la faun européenne. *Annales de La Société Entomologique de France*, *3*(5), 327–334.

Simon, E. (1875). *Les Arachnides de France. Tome seconde. Contenant les familles des Urocteidae, Agelenidae, Thomisidae et Sparassidae*. Roret.

Simon, E. (1876). Description d’araignées nouvelles de France. *Annales de La Société Entomologique de France*, *6*(5), 180–183.

Simon, E. (1882). II. Étude sur les arachnides de l’Yemen méridional: In Viaggio ad Assab nel Mar Rosso, dei signori G. Doria ed O. Beccari con il R. Aviso" Esploratore" dal 16 Novembre 1879 al 26 Febbraio 1880. *Annali Del Museu Civico Di Storia Naturale Di Genova*, *18*.

Simon, E. (1885). Matériaux pour servir à la faune arachnologiques de l’Asie méridionale. III. Arachnides recueillis en 1884 dans la presqu’île de Malacca, par M. J. Morgan. IV. Arachnides recueillis à Collegal, district de Coimbatoore, par M. A. Theobald G. R. *Bulletin de La Société Zoologique de France*, *10*, 436–462.

Simon, E. (1886a). Espèces et genres nouveaux de la famille des Thomisisdae. *Actes de La Société Linnéenne de Bordeaux*, *40*, 167–187.

Simon, E. (1886b). Etudes arachnologiques. 18e Mémoire. XXVI. Matériaux pour servir à la faune des Arachnides du Sénégal. (Suivi d’une appendice intitulé: Descriptions de plusieurs espèces africaines nouvelles). *Annales de la Société Entomologique de France (6)*, *5*, 345–396.

Simon, E. (1887). Arachnides. In *Mission scientifique du Cap Horn, 1882-1883* (Vol. 6, pp. E1–E42).

Simon, E. (1890). Etudes arachnologiques. 22e Mémoire. XXXIV. Etude sur les arachnides de l’Yemen. *Annales de La Société Entomologique de France*, *10*(6), 77–124.

Simon, E. (1897). Etudes arachnologiques. 27e memoire. XLII. Descriptions d’especes nouvelles de l’ordre des Araneae. *Annales de La Société Entomologique de France*, *65*, 465–510.

Simon, E. (1906). Voyage de M. Maurice Maindron dans l’Inde meridionale (mai a november 1901). 8me memoire. Arachnides (2e partie). *Annales de La Société Entomologique de France*, *75*(3), 279–314.

Simon, E. (1907). *Arachnides recueillis par L. Fea sur la côte occidentale d’Afrique: 1re partie*. Stabilimento tipo-litografico Pietro Pellas Fu La.

Simon, E. (1909). Etude sur les arachnides du Tonkin (1re partie). *Bulletin Scientifique de la France et de la Belgique*, *42*, 69–147.

Soares, B. A. M., & Soares, H. E. M. (1946). Contribuição ao estudo das aranhas do estado do Espírito Santo. *Papéis Avulsos Do Departamento de Zoologia*, *7*, 51–72.

Soares, B. M. (1943). Novos thomisidas brasilieros. *Papéis Avulsos Do Departamento de Zoologia, Secretaria de Agricultura, São Paulo*, *3*, 1–18.

Song, D. X. (1993). A new species of Thomisidae from China (Araneae). *Sinozoology*, *10*, 89–91.

Song, D. X., & Hu, J. (1986). A new species of Diaea from China (Araneae: Thomisidae). *Acta Zoológica Sínica*, *32*(4), 350–352.

Song, D. X., & Kim, J. P. (1992). A new species of crab spider from China, with description of a new genus (Araneae: Thomisidae). *Korean Arachnology*, *7*, 141–144.

Song, D. X., & Wang, X. P. (1994). Three new species of the family Thomisidae from Shaanxi, China (Araneae). *Acta Zoologica Sinica*, *19*, 46–50.

Song, D. X., & Zheng, S. X. (1981). A supplement note on three species of spiders from China. *Zoological Research*, *2*(4), 349–352.

Song, D. X., & Zhu, M. S. (1997). *Fauna sinica: Arachnida: Araneae, Thomisidae, Philodromidae*. Science Press.

Stellwag, L. M., & Dodson, G. N. (2010). Navigation by Male Crab Spiders Misumenoides formosipes (Araneae: Thomisidae): Floral Cues May Aid in Locating Potential Mates. *Journal of Insect Behavior*, *23*(3), 226–235. https://doi.org/10.1007/s10905-010-9209-9

Strand, E. (1907). Diagnosen neuer Spinnen aus Madagaskar und Sansibar. *Zoologischer Anzeiger*, *31*, 725–748.

Strand, E. (1913). Arachnida. I. In *Wissenschaftliche Ergebnisse der Deutschen Zentral-Afrika-Expedition 1907-1908, unter Führung Adolf Friedrichs, Herzogs zu Mecklenburg* (2; Vol. 4, pp. 325–474). Klinkhardt & Biermann.

Suman, T. W. (1971). Spiders of the family Thomisidae in Hawaii. *Pacific Insects*, *12*(4), 92.

Svatoň, J., Pekár, S., & Prídavka, R. (2000). Xysticus slovacus sp. n., a new thomisid spider from Slovakia (Araneae: Thomisidae). *Acta Universitatis Carolinae Biologica*, *44*(3), 157–162.

Szymkowiak, P. (2008). *Diaea Kangarooblaszaki* sp. Nov. from Kangaroo Island, South Australia (Araneae: Thomisidae). *Annales Zoologici*, *58*(2), 467–472. https://doi.org/10.3161/000345408X326799

Szymkowiak, P. (2014). Revision of Australian species of the genus Diaea (Araneae: Thomisidae) with redefinition of their taxonomic status. *Annales Zoologici*, *64*, Article 3.

Szymkowiak, P., & Dymek, A. (2012). A redefinition of the endemic Australian crab spider *Diaea Inornata* (L. Koch, 1876) (Araneae, Thomisidae). *New Zealand Journal of Zoology*, *39*(1), 57–69. https://doi.org/10.1080/03014223.2011.625956

Szymkowiak, P., & Królikowska, S. (2017). Redescription of Bomis larvata L. Koch, 1874 with the description of a new Australian species. *Zootaxa*, *4323*(4), 451. https://doi.org/10.11646/zootaxa.4323.4.1

Taczanowski, L. (1872). *Les Araneides de la Guyane française: Par Ladislas Taczanowski [Kopftitel.]*. Buchdruckerei W. Besobrasoff & Comp.

Tang, G., & Li, S. (2009a). Three new crab spiders from Xishuangbanna Rainforest, southwestern China (Araneae: Thomisidae). *Zootaxa*, *2109*(1), 45–58. https://doi.org/10.11646/zootaxa.2109.1.2

Tang, G., & Li, S. (2010a). Crab spiders from Hainan Island, China (Araneae, Thomisidae). *Zootaxa*, *2369*(1), 1. https://doi.org/10.11646/zootaxa.2369.1.1

Tang, G., & Li, S. (2010b). Crab spiders from Xishuangbanna, Yunnan Province, China (Araneae, Thomisidae). *Zootaxa*, *2703*(1), 1. https://doi.org/10.11646/zootaxa.2703.1.1

Tang, G., & Li, S.-Q. (2009b). The crab spiders of the Genus Tmarus from Xishuangbanna, Yunnan, China (Araneae: Thomisidae). *Zootaxa*, *2223*(1), 48–68. https://doi.org/10.11646/zootaxa.2223.1.3

Tang, G., LUO, W., & DENG, S. (2013). First description of the female Ozyptila kansuensis (Tang, Song & Zhu, 1995), comb. Nov.(Araneae: Thomisidae). *Zootaxa*, *3737*(1), 097–100.

Tang, G., Peng, X.-J., Griswold, C., Bick, D., & Yin, C.-M. (2008). Four crab spiders of the family Thomisidae (Araneae, Thomisidae) from Yunnan, China. 動物分類學報, *33*(2), 241–247.

Tang, G., Yin, C., Peng, X., & Griswold, C. E. (2009). Six crab spiders of the subfamily Stephanopinae from southeast Asia (Araneae: Thomisidae). *Raffles Bulletin of Zoology*, *57*(1), 39–50.

Tang, L. R., & Song, D.-X. (1988). On new species of the family Thomisidae from China (Arachnida: Araneae). *Acta Zootaxonomica Sinica*, *13*, 245–260.

Teixeira, R. A., & Barros, B. A. (2015). Taxonomic notes on the crab spider genera Stephanopoides and Isaloides (Araneae: Thomisidae: Stephanopinae). *Zootaxa*, *3956*(2), 281–287.

Teixeira, R. A., & Lise, A. A. (2012). Redescription of Misumenoides athleticus comb. Nov.(Araneae: Thomisidae), wrongly assigned to the philodromid genus Petrichus. *Zoologia (Curitiba, Brazil)*, *29*(4), 380–384.

Thaler, K. (1987). Drei bemerkenswerte Grossspinnen der Ostalpen (Arachnida, Aranei: Agelenidae, Thomisidae, Salticidae). *Mitteilungen Der Schweizerischen Entomologischen Gesellschaft*, *60*(3–4), 391–401. https://doi.org/10.5169/SEALS-402288

Thaler, K., & Zingerle, V. (1998). Ozyptila ladina n. Sp. From the Dolomites, northern Italy (Araneae, Thomisidae). *Bollettino Della Società Entomologica Italiana*, *130*, 99–104.

Thorell, T. (1875). Descriptions of several European and North-African spiders. *Kongl. Svenska Vet.-Akad. Handl.(NF)*, *13*, 3–203.

Thorell, T. (1877). Studi sui Ragni Malesi e Papuani. I. Ragni di Selebes raccolti nel 1874 dal Dott. O. Beccari. *Annali Del Museo Civico Di Storia Naturale Di Genova*, *10*, 341–637.

Thorell, T. (1881). Studi sui Ragni Malesi e Papuani. III. Ragni dell’Austro Malesia e del Capo York, conservati nel Museo civico di storia naturale di Genova. *Annali Del Museo Civico Di Storia Naturale Di Genova*, *17*, 1–720.

Thorell, T. (1887). Viaggio di L. Fea in Birmania e regioni vicine. II. Primo saggio sui ragni birmani. *Annali Del Museo Civico Di Storia Naturale Di Genova*, *25*, 5–417.

Thorell, T. (1891). Spindlar från Nikobarerna och andra delar af södra Asien. *Kongliga Svenska Vetenskaps-Akademiens Handlingar*, *24*(2), 1–149.

Thorell, T. (1894). Decas aranearum in ins. Singapore a Cel. Th. Workman inventarum. *Bullettino Della Società Entomologica Italiana*, *26*, 321–355.

Thorell, T. (1895). *Descriptive catalogue of the spiders of Burma, based upon the collection made by Eugene W. Oates and preserved in the British Museum.* Printed by order of the Trustees,. https://doi.org/10.5962/bhl.title.7163

Tian, T., Zhou, G.-C., & Peng, X.-J. (2018). A new species of Pistius Simon, 1875 (Araneae: Thomisidae) from Wuling Mountains, China. *Acta Arachnologica Sinica*, *27*(1), 5.

Tikader, B. K. (1961). On two new species of spider of the genus Oxyptila (family Thomisidae) from India. *Proceedings of the Zoological Society, Calcutta*, *13*, 115–118.

Tikader, B. K. (1980). *Araneae: Pt. 1. Thomisidae (Crab-spiders)* (Vol. 1). Zoological Survey of India.

Tripathi, R., Jangid, A. K., Bhagirathan, U., & Sudhikumar, A. V. (2023). First record of the genus *Bassaniodes* Pocock, 1903 (Araneae, Thomisidae) from India. *Natura Somogyiensis*, *40*, 47–50.

Tullgren, A. (1905). Araneida from the Swedish expedition through the Gran Chaco and the Cordilleras. *Arkiv För Zoologi*, *2*(19), 1–81.

Urones, C. (1998). Descripción de Oxyptila bejarana n. Sp. De la Sierra de Béjar (Salamanca, España) (Araneae, Thomisidae). *Revue Arachnologique*, *12*, 79–88.

Utochkin, A. S., & Savelyeva, L. G. (1995). Review of the spider genus Xysticus C. L. Koch, 1835 (Arachnida Aranei Thomisidae) in the East Kazakhstan area. *Arthropoda Selecta*, *4*(1), 65–69.

van Helsdingen, P. (1986). Discrimination between Xysticus luctuosus (Blackwall) and X. Acerbus Thorell (Araneae, Thomisidae). *Mémoires de La Société Royal Belge d’Entomologie*, *33*, 85–92.

van Niekerk, P., & Dippenaar-Schoeman, A. S. (2013). A Revision of the Crab Spider Genus *Heriaeus* Simon, 1875 (Araneae: Thomisidae) in the Afrotropical Region. *African Invertebrates*, *54*(2), 447–476. https://doi.org/10.5733/afin.054.0213

Vieira, C. (2015). *Evolução de fluorescência, cripsia e comportamentos em aranhas Thomisidae sobre flores* [Universidade Estadual de Campinas]. http://repositorio.unicamp.br/jspui/handle/REPOSIP/315934

Vieira, C., Ramires, E. N., Vasconcellos-Neto, J., Poppi, R. J., & Romero, G. Q. (2017). Crab Spider Lures Prey In Flowerless Neighborhoods. *Scientific Reports*, *7*(1), 9188. https://doi.org/10.1038/s41598-017-09456-y

Wang, C., Mi, X., & Peng, X. (2016). A new species of Pharta Thorell, 1891 (Araneae: Thomisidae) from China. *Oriental Insects*, *50*(3), 129–134. https://doi.org/10.1080/00305316.2016.1197163

Wang, X. G., & Xi, G. S. (1998). A new species of the genus Tmarus from Shaanxi Province, China (Araneae: Thomisidae). *Acta Arachnologica Sinica*, *7*(1), 33–35.

Workman, T. (1896). *Malaysian spiders: Vol. I*.

Wunderlich, J. (1987). *Die Spinnen der Kanarischen Inseln und Madeiras: Adaptive Radiation, Biogeographie, Revisionen und Neubeschreibungen* (1). Triops.

Wunderlich, J. (1995). Zur Kenntnis west-paläarktischer Arten der Gattungen *Psammitis* Menge 1875, *Xysticus* C. L. Koch 1835 und *Ozyptila* Simon 1864 (Arachnida: Araneae: Thomisidae). *Beiträge Zur Araneologie*, *4*(1994), 749–774.

Wunderlich, J., & Schultz, W. (1995). Ozyptila westringi (Thorell 1873), eine in Deutschland seltene Krabbenspinnenart (Arachnida: Araneae: Thomisidae). *Beiträge zur Araneologie*, *4*, 329–333.

Yang, Z. Z., Zhu, M. S., & Song, D. X. (2006). A newly recorded genus from China and two new species of the family Thomisidae (Arachnida Araneae). *Acta Arachnol. Sin.*, *15*, 65–69.

Yang, Z.-Z., Zhu, M.-S., & Song, D.-X. (2005). Two new species of the spider genus Tmarus Simon 1875 (Araneae: Thomisidae) from China. *Acta Arachnologica*, *54*(2), 95–98.

Yang, Z.-Z., Zhu, M.-S., & Song, D.-X. (2006). A new species of the genus Sanmenia Song & Kim, 1992 (Araneae, Thomisidae) from Yunnan Province, China. *Zootaxa*, *1151*(1), 41. https://doi.org/10.11646/zootaxa.1151.1.3

Yin, C. M., Peng, X. J., Yan, H. M., Bao, Y. H., Xu, X., Tang, G., Zhou, Q. S., & Liu, P. (2012). *Fauna Hunan: Araneae in Hunan, China* (Vol. 1590). Hunan Science and Technology Press.

Yin, C. M., Peng, X., & Kim, J. (1999). Three new species of the genus Philodromus from China (Araneae: Philodromidae). *Korean Journal of Biological Sciences*, *3*(4), 355–358. https://doi.org/10.1080/12265071.1999.9647507

Yu, L., Xu, X., Zhang, Z., Painting, C. J., Yang, X., & Li, D. (2022). Masquerading predators deceive prey by aggressively mimicking bird droppings in a crab spider. *Current Zoology*, *68*(3), 325–334. https://doi.org/10.1093/cz/zoab060

Yuan, T., Niu, C. L., Ye, X. Y., & Zhang, Z. S. (2019). A newly recorded crab-spider Monaeses israeliensis (Thomisidae) from Xinjiang, China. *Acta Arachnologica Sinica*, *28*(2), 106–108.

Zamani, A., & Marusik, Y. M. (2017). Six new species of spiders (Arachnida: Araneae) from Iran. *Oriental Insects*, *51*(4), 313–329. https://doi.org/10.1080/00305316.2017.1282386

Zhang, J.-X., Zhu, M.-S., & Tso, I.-M. (2006). Four new crab spiders from Taiwan (Araneae, Thomisidae). *Journal of Arachnology*, *34*(1), 77–86. https://doi.org/10.1636/H04-99.1

Zhang, Y. J., & Yin, C. M. (1998). A new species of the genus Oxytate from China (Araneae: Thomisidae). *Dong Wu Fen Lei Xue Bao= Acta Zootaxonomica Sinica*, *23*(1), 6–8.

Zhu, M. S., & Shan, Y. J. (2007). The new discovery of the female spider Stiphropus ocellatus Thorell, 1887 from China (Araneae: Thomisidae). *Acta Zootaxo. Sin.*, *32*, 913–914.

Zhu, M. S., & Song, D. X. (2006). A new discovery of the male spider and a new record from China (Araneae, Thomisidae). *Acta Zootaxonomica Sinica*, *31*, 549–552.

Zuo, W.-X., Guo, C.-H., & Zhang, F. (2014). A newly recorded species of the genus Xysticus (Araneae: Thomisidae) from China. *Acta Arachnologica Sinica*, *23*(2), 4.
